# Intrinsic changes in cell differentiation and identity drive impaired wound healing in aged female murine skin

**DOI:** 10.1007/s10522-025-10340-w

**Published:** 2025-11-01

**Authors:** Christabel Thembela Dube, Gokce Oguz, Yasmin Hui Binn Ong, Samydurai Sudhagar, Shyam Prabhakar, Matthew Ronshaugen, Adaikalavan Ramasamy, Chin Yan Lim, Kimberly A. Mace

**Affiliations:** 1 A*STAR Skin Research Labs (A*SRL), Agency for Science, Technology and Research (A*STAR), 8 A Biomedical Grove, #06-06 Immunos, Singapore, 138648 Republic of Singapore; 2 School of Biological Sciences, Faculty of Biology, Medicine and Health, University of Manchester, Manchester, M13 9PT UK; 3 Genome Institute of Singapore (GIS), Agency for Science, Technology and Research (A*STAR), 60 Biopolis Street, Genome #02-01, Singapore, 138632 Republic of Singapore; 4 Department of Biochemistry, Yong Loo Lin School of Medicine, National University of Singapore, Singapore, 117597 Republic of Singapore

**Keywords:** Ageing, Wound healing, Skin, Macrophages, Keratinocytes, Single-cell RNA-sequencing

## Abstract

Cellular and molecular mechanisms that drive a perturbed wound microenvironment and impaired healing in aged skin have not been fully delineated. To obtain a comprehensive understanding of cell-intrinsic changes acquired during ageing that impact early responses to injury, we performed single-cell RNA sequencing in young and aged intact female murine skin and wounds 3 days post-injury. We observed that substantial changes in the mean proportional distribution and transcriptomic state of skin resident subpopulations in aged, but not young, tissues accompany a global increase in basal inflammation. This is driven by an altered signalling environment leading to impaired keratinocyte differentiation, loss of fibroblast identity and defective macrophage function. Further, we show that ageing-induced changes in skin resident cells persist after injury, resulting in increased expression of senescence-related genes in wound fibroblasts and aberrant monocyte-to-macrophage transitioning coupled to an enhanced inflammatory signature and defective intercellular signalling in comparison to wounds in young mice. In summary, our data highlights a contribution of both cell-intrinsic changes and an altered tissue microenvironment to poor wound healing responses in aged mice.

**Supplementary Information:**

The online version contains supplementary material available at 10.1007/s10522-025-10340-w.

## Introduction

Early cellular events are critical in determining the overall success of wound healing. Following injury, coordinated interactions between skin resident cells such as keratinocytes, fibroblasts, macrophages and endothelial cells, promote optimal wound healing progression (Guerrero-Juarez et al. 2019; Haensel et al. 2020; Phan et al. 2021). Healing begins with haemostasis where vasoconstriction reduces blood flow and red blood cells aggregate with platelets to form a plug. Degranulating platelets then release chemokines and cytokines that activate tissue-resident macrophages and keratinocytes (Sonmez & Sonmez 2017; Xia & Kao 2003). Upon activation, resident macrophages and keratinocytes secrete additional proinflammatory cytokines to initiate the inflammatory phase of wound healing (Barker et al. 1991; de Oliveira et al. 2013; Kemény et al. 1994). Neutrophils migrate into the tissue within 12 hours, and remain at peak levels, clearing tissue debris for several days (Kim et al. 2008). Approximately 1–2 days after injury, CC motif chemokine receptor 2 (CCR2) expressing monocytes are recruited to the wound where they differentiate into macrophages (Boniakowski et al. 2018). Monocyte-derived macrophages play a pivotal role in all phases of injury, and importantly, promote the transition from the early inflammatory responses to the proliferative and remodelling phases of healing. During this transition, keratinocytes continue to proliferate and migrate to reepithelialise the skin while macrophages secrete pro-healing growth factors, vascular endothelial growth factor (VEGF) and transforming growth factor-beta 1 (TGF-β1) that drive fibroblast proliferation and granulation tissue formation (Haensel et al. 2020; Landén et al. 2016).

Delayed wound closure in aged skin is characterised by impaired progression of healing responses resulting in poor reepithelialisation, extended inflammatory responses and delayed granulation tissue formation. Aged keratinocytes in Day 3 and Day 5 mouse wounds were found to migrate slower and exhibit impaired cellular interactions with immune cells in comparison to young keratinocytes (Keyes et al. 2016). Moreover, macrophage distribution was altered in aged but not young Day 3 wounds. However, Day 7 wounds did not exhibit any differences in macrophage proportions between young and aged tissues indicating that early healing responses were the most affected by ageing (Hardman & Ashcroft 2008; Swift et al. 2001). Poor inflammation resolution and increased tissue damage in aged Day 3 wounds were associated with hyperinflammatory macrophages with a reduced proliferative capacity (Dube et al. 2022a). These studies demonstrate that healing responses to wounding are impaired in aged murine tissues. While there are significant differences in murine cutaneous wound healing compared to human, such as a dependence on wound contraction in mouse skin, many wound healing processes, cell types and gene expression changes are conserved between them, making aged mice a useful model to explore changes in cell fate dynamics (Khalid et al. 2022; Wang et al. 2024).

To gain a comprehensive understanding of cell-intrinsic characteristics acquired upon ageing that impact early responses to wounds, we performed single-cell RNA sequencing of 78,670 cells from young (3 months) and aged (22–24 months) female mouse skin and wounds 3 days post-injury. First, we show that ageing drives intrinsic transcriptomic alterations in the resident epidermal, fibroblasts and macrophage cell populations in the skin resulting in impaired function. Second, we demonstrate dysregulated wound fibroblast function, altered neutrophil subpopulation balance and disrupted monocyte-macrophage transitioning patterns underlie impaired early healing responses in aged wounds. Finally, we delineate global signalling networks associated with defective transcriptomic regulation in the aged wound microenvironment, revealing potential targets for restoring optimal wound repair. In summary, our data propose a synergistic contribution of cell-intrinsic changes and an altered tissue microenvironment to ageing-induced poor wound healing responses.

## Results

### Ageing-related changes in the distribution of cell populations in intact skin and wounded tissues

**Fig. 1:**
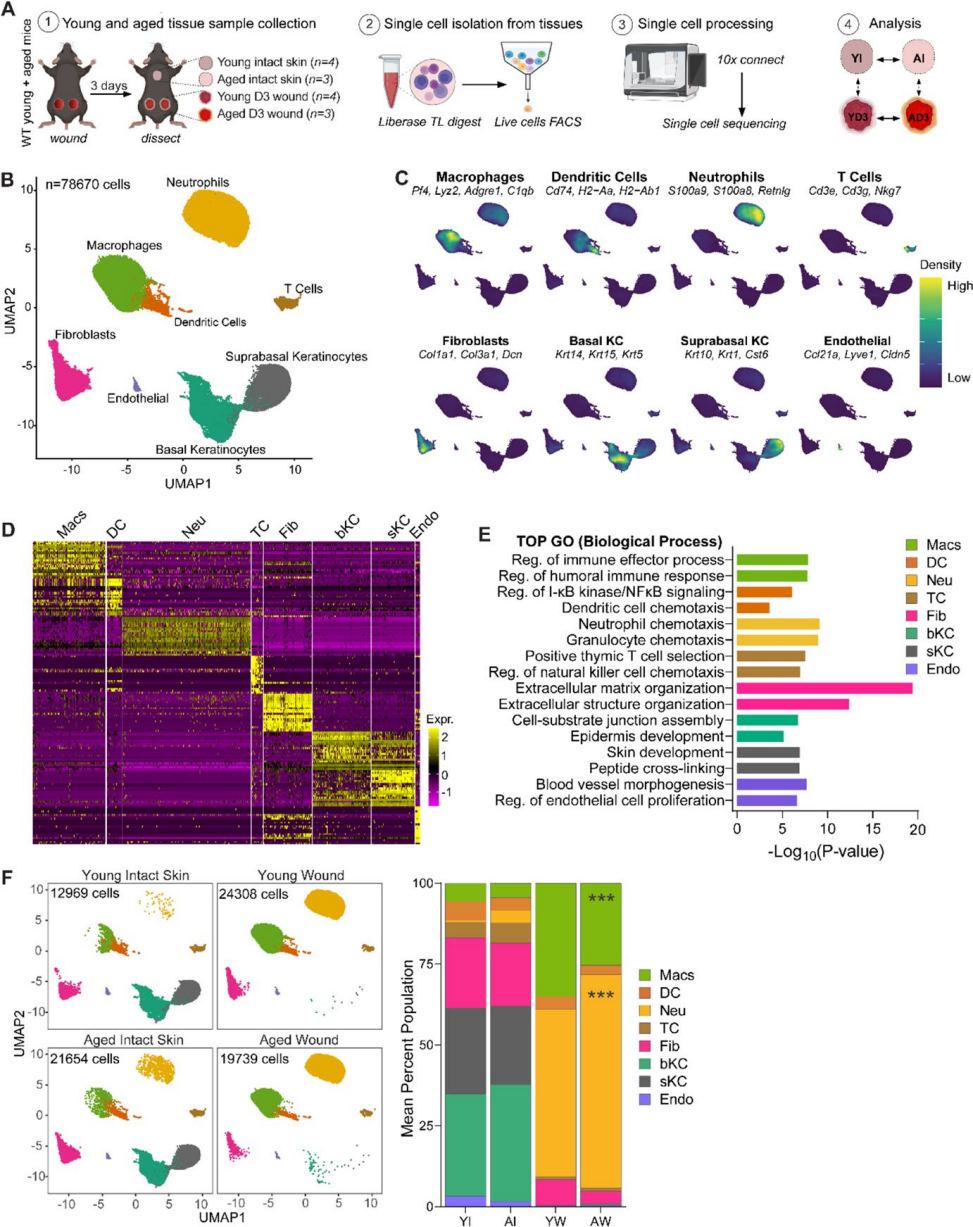
Single-cell analyses of skin resident cell populations in the skin and during early wound healing responses in young and aged mice**. A** Experimental design for single-cell experiments. Intact skin and Day 3 wounds were excised from young (3 months) and aged (22–24 months) C57BL6/J wild-type (WT) female mice and subjected to single-cell RNA sequencing on the 10X Chromium Connect platform. *n* = 3–4 mice per group. **B** UMAP illustrating identified major cell types in young and mouse skin and wounds (*n* = 78,670 cells). **C** Visualisation of the distribution of cell type-specific markers in each population. **D** Heatmap showing the top 20 differentially expressed genes in each major cell type. **E** Enriched gene ontology biological processes in major cell types identified in young and aged skin and wound tissues. **F** (left) Sample type stratified UMAP visualisation of the distribution of major cell types in young intact skin, aged intact skin, young wounds and aged wounds. (right) Mean percentage population of identified cell types in young and aged intact skin and wounds. *n* = 3–4 mice per group. ***p < 0.001, two-way ANOVA with a Tukey multiple comparisons test. *GO* Gene ontology*, Mac* macrophages*, Neu* neutrophils, *DC* dendritic cells, *TC* T cells, *FB* fibroblasts, *bKC* basal keratinocytes, *sKC* suprabasal keratinocytes, *Endo* endothelial cells, *YI* young intact skin, *AI* aged intact skin, *YW* young wound (day 3), *AW* aged wound (day 3)

To investigate cell-intrinsic characteristics of aged skin that impact early responses to injury, we isolated live single cells from intact skin and Day 3 wounds from young (3 months) and aged (22–24 months) wildtype C57BL6/J mice and performed single-cell RNA sequencing Fig. 1A. Following rigorous quality control, we obtained 78,760 cells for further analysis Fig. 1B, Fig S1A, Table S1. Cells were clustered into 8 major cell types: macrophages (Macs, expressing Pf4, Lyz2, Adgre1), dendritic cells (DC, expressing H2-Ab1, Cd74, H2-Eb1), neutrophils (Neu, expressing S100a9, S100a8, Retnlg), T cells (TC, expressing Cd3g, Cd7, Nkg7), fibroblasts (FB, expressing Col1a1, Col3a1, Dcn), basal keratinocytes (bKC, expressing Krt14, Krt15, Krt5), suprabasal keratinocytes (sKC, expressing Krt10, Krt1, Cst6), and endothelial cells (Endo, expressing Pecam1, Lyve1, Cldn5). Figure 1B-D, Fig S1B-C, Supplementary File 2. The most enriched biological process gene ontology (GO) terms in each cell type were consistent with their known biological functions Fig. 1E.

We analysed cell proportions in the young and aged intact and wounded tissues for ageing-related differences Fig. 1F. In both young and aged skin, keratinocytes were the most abundant population, with aged skin having a slightly higher percentage of basal KC (36%) compared to young skin (31%). Upon wounding, both KC populations were ablated in both young and aged wounds due to loss of the epidermis. Fibroblasts, the second most abundant population in the skin, were marginally reduced in aged skin tissues (19%) in comparison to young (23%) Fig. 1F. Following wounding, we observed a decrease in the population in both young and aged tissues. Consistent with our previous study, the proportion of FBs was 50% lower in aged compared to young Day 3 wounds (Dube et al. 2022a). Myeloid cells, which include macrophages, dendritic cells and neutrophils, were the lowest population in normal skin. The proportion of macrophages and DCs was lower in aged skin. As expected, neutrophils were almost absent in the young tissues, comprising less than 1% of the total skin cell population. However, we noted a higher number of neutrophils in aged skin, encompassing 4% of the total aged skin cells Fig. 1F. In Day 3 wounds, there was a sharp increase in the neutrophil and macrophage populations signifying the early inflammatory response Fig. 1F. The proportion of neutrophils in aged wounds was 14% higher than in young wounded tissues. By contrast, aged wounds exhibited 10% fewer macrophages in comparison to young wounds. This data confirmed an altered expansion of neutrophils and macrophages in response to inflammation in aged wounds. Taken together, these results demonstrate that ageing has minor effects on the cellular composition at steady state conditions but significantly alters population distributions of myeloid cells during the early inflammatory response to wounding.

### Ageing-associated changes in proliferative basal cells impact keratinocyte differentiation

**Fig. 2:**
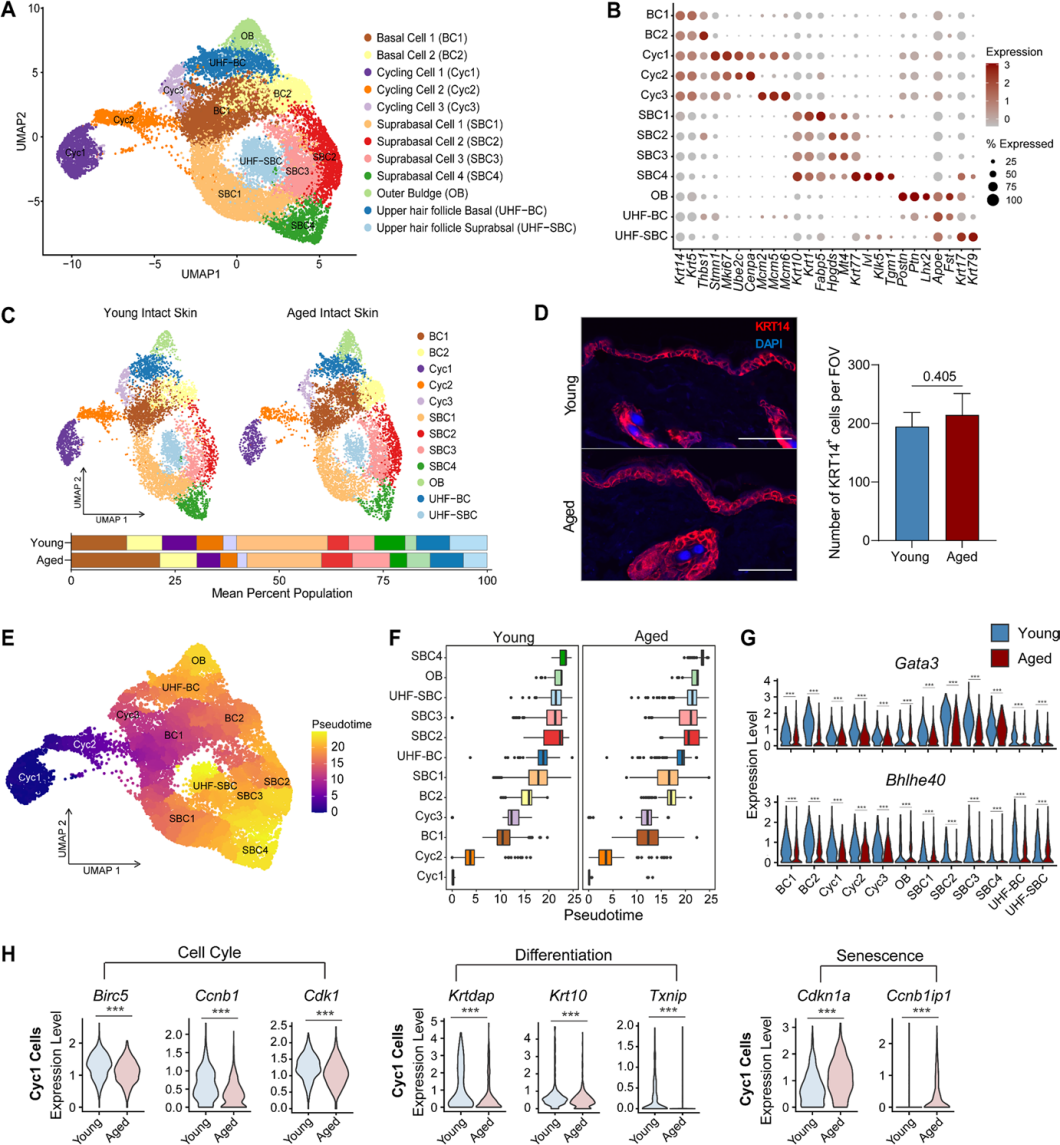
Intrinsic changes in proliferative basal cells impact keratinocyte differentiation trajectories in aged mice**. A** UMAP visualisation of subclustered basal and suprabasal keratinocyte populations in young and aged skin. **B** Average expression of keratinocyte subpopulation markers. **C** UMAP visualisation of differences in the distribution of keratinocyte cell type population in young and aged intact skin. Average percentage population of identified keratinocyte cell types in young and aged skin (Two-way ANOVA with a Bonferroni multiple comparisons test). **D** Representative confocal images of KRT14 staining in young and aged mouse skin. Scale bar = 50 µm. Bar chart shows the number of KRT14.^+^ cells per field of view (FOV) in young and aged skin (*n* = 4, two-tailed unpaired *T*-test, Cohen’s d effect size = 0.66, 95% Confidence Interval: −34.17 to 73.59. Data are shown as mean ± SD)**. E** UMAP coloured by pseudotime showing analyses of keratinocyte differentiation trajectories using Monocle3. **F** Boxplot showing differences in keratinocyte population differentiation dynamics in young and aged skin along a pseudotime. **G** Violin plots of gene expression levels of *Gata3* and *Bhlhe40* in young and aged keratinocyte populations (***p < 0.001, Two-tailed unpaired *T*-test). **H** Violin plots of gene expression levels of *Birc5, Ccnb1, Cdk1, Krtdap, Krt10, Txnip, Cdkn1a,* and *Ccnb1ip1* in young and aged Cyc1 cells (***p < 0.001, Two-tailed unpaired *T*-test)

To assess the effect of ageing on epidermal barrier function during homeostasis, we aggregated two major epidermal cell types, basal and suprabasal KCs from young and aged skin (20,773 cells), performed further sub-clustering and annotated 12 epidermal cell types defined by their expression of canonical markers previously validated in publicly available datasets Fig. 2A–B (Cheng et al. 2018; Hu et al. 2021; Joost et al. 2016). We defined 5 subpopulations

expressing high levels of basal KC markers *Krt14* and *Krt5* and termed them Basal Cells 1 and 2 (BC1 and BC2) and Cycling Cells 1,2, and 3 Fig. 2A–C. BC1 had the highest expression of *Krt14* and *Krt5,* while BC2 uniquely expressed high levels of *Thbs1*, a glycoprotein involved in cellular interactions with the extracellular matrix (Siriwach et al. 2022). Cyc1, Cyc2, and Cyc3 cells expressed high levels of proliferation markers including *Mki67* and *Stmn1,* suggesting that they represent a proliferative epidermal transit amplifying subpopulation of *Krt14*^*hi*^ cells Fig. 2A–B. We also identified four suprabasal cell populations, which we termed SBC1, SBC2, SBC3 and SBC4, exclusively expressing high *Krt10* and *Krt1*, with SBC4 exhibiting increased levels of *Krt77*, *Ivl* and *Klk5* indicative of a terminally differentiated state. We also defined 3 hair follicle-associated populations, outer bulge (OB, high *Lhx2*, *Postn*, *Ptn*), basal upper hair follicle cells (UHF-BC, high *Apoe*, *Fst*) and suprabasal upper hair follicle cells (UHF-SBC, high *Krt17* and *Krt79*, *Apoe*) Fig. 2A–B, Supplementary File 3.

Cell distribution analyses revealed differences in the proportions of 5 identified basal KC subpopulations (BC1, BC2, Cyc1, Cyc2 and Cyc3), despite no changes in the total number of *Krt14*^*hi*^ cells in young and aged skin Fig. 2C–D. We found that aged intact skin had higher mean proportions of BC1 and BC2 but diminished Cyc1, Cyc2 and Cyc3 proliferating populations in comparison to young Fig. 2C. This data suggested that there was an expansion of the BC1 and BC2 population, but it was unlikely to be due to increased proliferation, as there were fewer proliferating KCs in the aged skin. Aged tissues also exhibited reduced amounts of SBC1 and SBC4 *Krt10*^*hi*^ KCs, suggestive of ageing-associated alterations in KC differentiation dynamics resulting in impaired transition from basal to suprabasal states. To evaluate if the differentiation of KCs was altered with age, we performed pseudotime trajectory analyses using *Monocle3*. By setting Cyc1 as the root population, we observed that both young and aged epidermal cell clusters followed the expected differentiation trajectories where basal and transit amplifying cells appeared at the beginning of the trajectory and differentiated suprabasal cell types were placed later along the pseudotime Fig. 2E–F. However, significant temporal differences in the differentiation of young and aged epidermal cells were observed Fig. 2F. Gene expression analysis further revealed transcription factors known to regulate KC differentiation, such as *Gata3* and *Bhlhe40*, were markedly reduced in all aged epidermal cell types in comparison to young Fig. 2G. Furthermore, we found that aged Cyc1 cells had reduced levels of cell-cycle-related *Birc5*, *Ccnb1* and *Cdk1* and differentiation-related *Krtdap, Krt10* and *Txnip* but expressed high levels of senescence-related *Cdkn1a* and *Ccnba1ip1* compared to Cyc1 cells from young skin Fig. 2H. Collectively, our data highlights a compromised epidermis in aged mice resulting from altered proliferation and differentiation of the keratinocytes.

### Altered fibroblast function in aged skin is associated with impaired wound healing responses

**Fig. 3:**
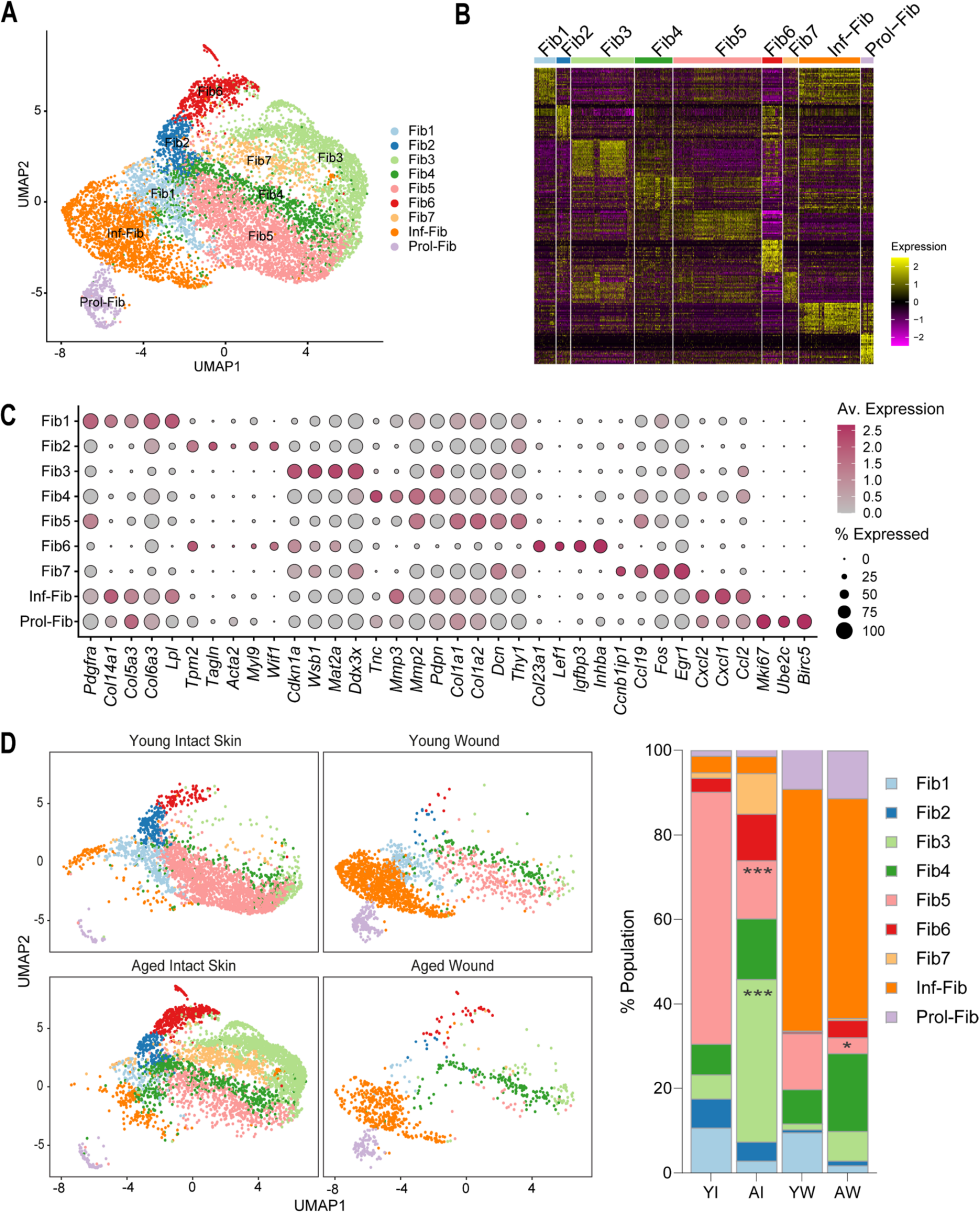
Fibroblast distribution in young and aged skin and wounds. **A** UMAP visualisation of 9 defined fibroblast subtypes identified in young and aged skin and wounds. **B** Heatmap of top 20 differentially expressed genes in each of the fibroblast subpopulations. **C** Dot plot of the average expression of distinguishing top markers in fibroblast subpopulations. **D (left)** UMAP visualisation of fibroblasts segregated by sample type to highlight separation or overlap between young and aged fibroblast groups in intact skin or wounds.** (right)** Mean percentage population of identified fibroblast populations in young and aged intact skin and wounds. *n* = 3–4 mice per group. *p < 0.05, ***p < 0.001, two-way ANOVA with a Tukey multiple comparisons test. *Fib* fibroblasts, *Inf-fib* inflammatory fibroblasts, *Prol-fib* proliferating fibroblasts, *Av* average

To examine the influence of ageing on the dermis, we subclassified 9,918 fibroblast cells (FB) from young and aged skin and wounds into 9 defined cell populations. The defined population clusters were Fib1 (*Col6a3*^*hi*^), Fib2 (*Tpm2*^*hi*^), Fib3 (*Cdkn1a*^*hi*^), Fib4 (*Tnc*^*hi*^), Fib5 (*Col1a1*^*hi*^), Fib6 (*Col23a1*^*hi*^), Fib7 (*Egr1*^*hi*^), inflammatory fibroblasts (*Cxcl1*^*hi*^) and proliferating fibroblasts (*Mki67*^*hi*^) Fig. 3A–C, Supplementary File 4. We found that specific clusters were differentially associated with young or aged skin. Fib1 and Fib5 were enriched in young but not aged tissues and expressed high levels of genes associated with extracellular matrix and collagen organisation. By contrast, Fib3, 4, 6 and 7 were associated with aged skin and exhibited loss of canonical FB function Fig. 3C–D, Fig S2A. Immunostaining analyses revealed a reduction in the number of pro-collagen type I expressing cells in aged dermis, indicative of reduced collagen production and extracellular matrix protein synthesis in aged skin Fig. S2B. These observations were consistent with previous studies that described increased transcriptional variability and ‘identity noise’, in which cells display poor expression of canonical FB markers in aged FBs (Almet et al. 2024; Mahmoudi et al. 2019; Salzer et al. 2018; Solé-Boldo et al. 2020).

**Fig. 4:**
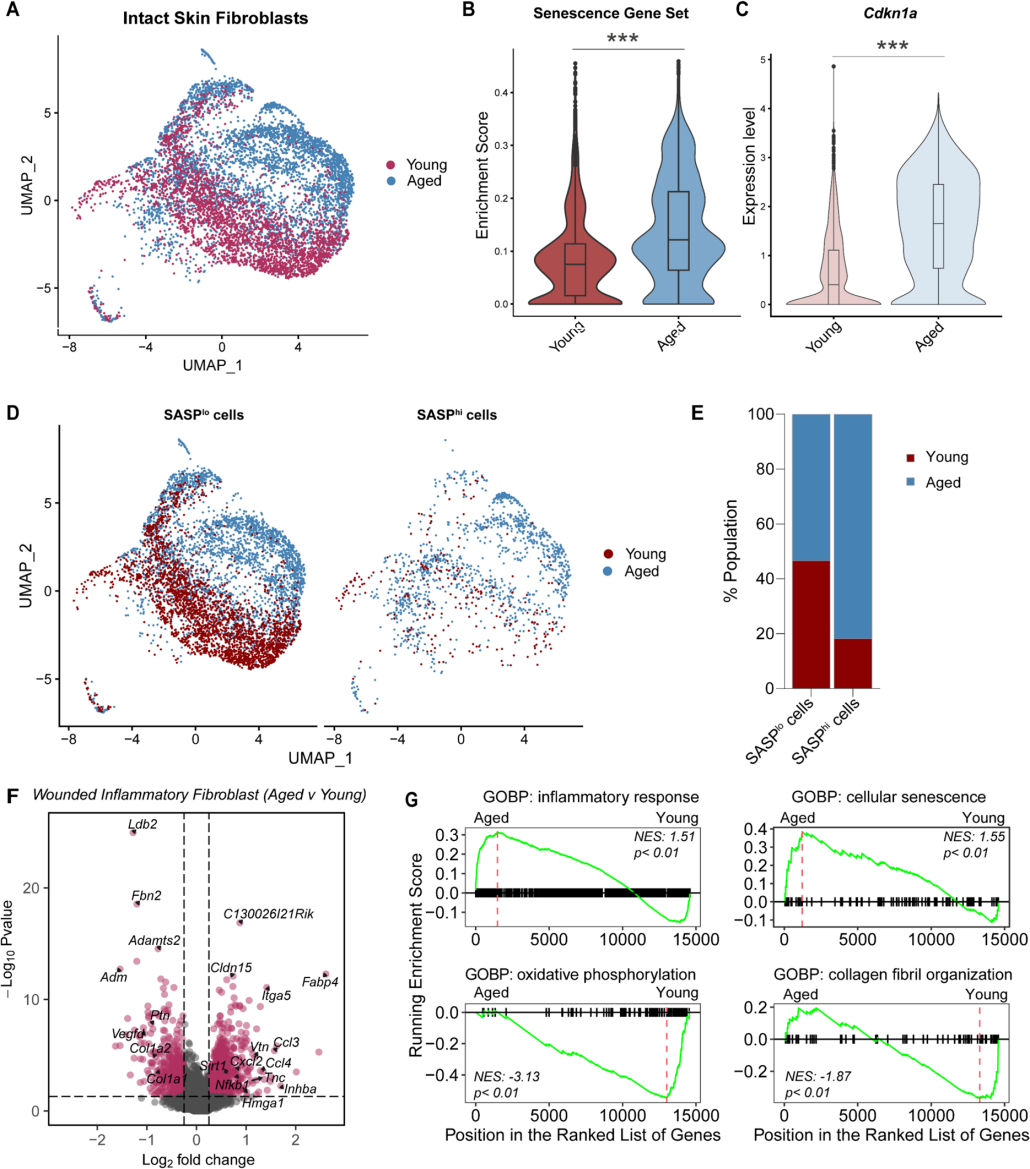
Fibroblast function is impaired in aged skin and wounds: **A** UMAP visualisation of fibroblasts in young and aged intact skin **B** Violin plots displaying enrichment score for the SenMayo senescence geneset in young and aged fibroblasts (***p < 0.001, Two-tailed unpaired *T*-test)**. C** Violin plot showing the expression level of Cdkn1a in young and aged fibroblasts (***p < 0.001, Two-tailed unpaired *T*-test). **D** UMAP visualisation of the distribution of SASP^hi^ and SASP^lo^ cells in young and aged fibroblasts in intact skin. **E** Bar chart of the percentage proportion of SASP^lo^ versus SASP^hi^ fibroblasts in young and aged skin. **F** Volcano plot illustrating differentially expressed genes in aged versus young inflammatory fibroblasts in Day 3 wounds (adjusted *p*-value < 0.05, log2 foldchange > 0.25, data analysed using DESeq2). **G** Gene set enrichment analyses of gene ontology biological processes (GOBP) altered in aged versus young wound inflammatory fibroblasts. In each plot, gene sets, represented by vertical hatches across the horizontal black line, are ranked from the most to the least enriched in aged versus young wound inflammatory fibroblasts. The curve (green line) represents the running enrichment score of the ranked gene list. The maximum enrichment scores in each set are indicated by the red dashed line. Normalised enrichment score (NES) and *p*-value are shown for each gene set. Inflammatory response and cellular senescence are upregulated while oxidative phosphorylation and collagen fibril organisation are downregulated in aged wound fibroblasts in comparison to young

The FBs from young and aged skin formed separate cell clusters Fig. 4A. To further evaluate ageing-related differences, we assessed the enrichment of senescence-related genes, based on the *SenMayo* geneset of 125 senescence-associated-secretory phenotype (SASP) factors (Saul et al 2022), in the young and aged fibroblast populations in intact skin. We found that aged FBs had significantly higher enrichment scores and elevated expression of senescence-associated genes, such as *Cdkn1a* (p21), in comparison to young FBs Fig. 4B–C. Aged skin also had a higher proportion of SASP^hi^ FBs (81.8%) in comparison to young, suggesting a more pronounced senescence-related phenotype Fig. 4D–E.

Next, we assessed how the altered aged FBs influenced wound healing responses. Following wounding, we found that young and aged mice responded similarly by expanding the inflammatory FB population, but the mean proportion of inflammatory FB was reduced in aged wounds Fig. 3C–D. Interestingly, unlike in skin, we found no age-related differences in cellular composition in wounds. We compared the transcriptomes of inflammatory FBs in young versus aged wounds and found that 145 genes were differentially expressed (adj_*p* < 0.05, log2 foldchange > 0.25, Fig. 4F, Supplementary File 4. Inflammatory cytokines *Ccl4*, *Ccl3*, and *Cxcl2* were upregulated in aged inflammatory FBs indicating enhanced inflammatory signatures in these cells, while genes associated with collagen fibril organisation such as *Col1a2* and *Col1a1* were downregulated Fig. 4F. Gene set enrichment analyses further confirmed the upregulation of inflammation and senescence, and downregulation of collagen fibril organisation and oxidative phosphorylation pathways in aged inflammatory FBs Fig. 4G. Together, these results suggest that aged FBs have altered responses to wounding that contribute to impaired wound closure in aged tissues.

### Functionally defined neutrophil subpopulations are differentially enriched in young and aged tissues

**Fig. 5:**
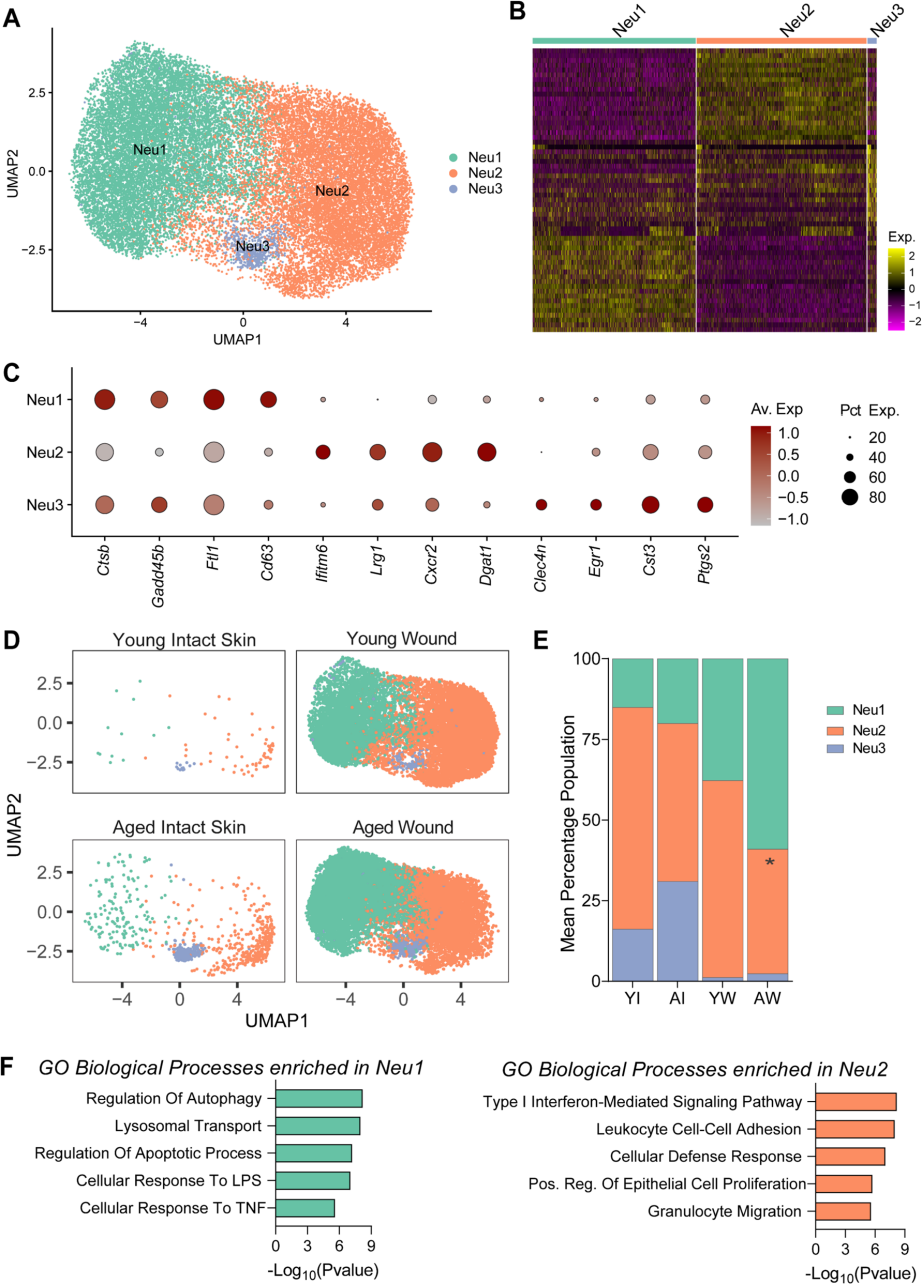
Defined neutrophil subpopulations are differentially enriched in young and aged wounded tissues**. A** UMAP visualisation of neutrophil subpopulation in young and aged tissues. **B** Heatmap of top 20 differentially expressed genes in defined neutrophil subpopulations. **C** Dot plot of the average expression of distinguishing top markers in neutrophil subpopulations. **D** UMAP visualisation of the distribution of neutrophils subtypes in young and aged intact skin and Day 3 wounded tissues. **E** Mean percentage distribution of Neu1, Neu2 and Neu3 cells in young and aged intact skin and wounds *n* = 3–4 mice per group. *p < 0.05, two-way ANOVA with a Tukey multiple comparisons test. **F** Enriched GO terms (biological processes) in neutrophil subsets Neu1 and Neu2 in young and aged wounds. **Abbreviations**: *GO:* gene ontology*, Neu:* neutrophils*, YI:* young intact skin*, AI:* aged intact skin*, YW:* young wound (day 3)*, AW:* aged wound (day 3)

Next, we assessed the impact of young and ageing immune cells in early responses to wounding. Transcriptomic clustering of 26,502 neutrophils from young and aged tissues uncovered three defined subpopulations we termed Neu1, Neu2 and Neu3 Fig. 5A–B, Supplementary File 5. Neu1 cells expressed high levels of *Ctsb* and *Cd63* involved in neutrophil activation. Neu2 cells expressed high *Ifitm6* and *Lrg1,* associated with type 1 interferon responses and neutrophil differentiation. Neu3 was the smallest cluster expressing elevated levels of *Egr1*, *Cts3* as well as inflammation-associated gene *Ptgs2* Fig. 5A–C, Supplementary File 5.

Upon wounding, Neu1 and Neu2 cell populations were markedly increased in both young and aged tissues, with aged wounds having a higher proportion of Neu1 cells (64.5%) while Neu2 made up 60% of the neutrophil population in young wounds Fig. 5D–E. Gene ontology analysis on differentially expressed genes between Neu1 and Neu2 populations (Supplementary File 5, *p* < 0.05, log2 fold change > 1) showed that Neu1 cells expressed genes associated with regulation of autophagy, lysosomal transport, and response to LPS and TNF. Conversely, Neu2 cells highly expressed genes involved in the type1 interferon signalling pathway, cell defence response and positive regulation of epithelial cell proliferation during wound healing Fig. 5F.

We compared the Neu1 and Neu2 cell populations in young and aged wounds to uncover any ageing-associated alterations in these cells. We found 131 genes that were significantly differentially expressed in aged Neu1 cells, while 102 genes were changed in aged Neu2 cells in comparison to young (adjusted *p*-value < 0.05, log2 foldchange > 0.25, Fig S3A & S3C, Supplementary File 5). Aged Neu1 cells displayed upregulation of inflammatory response and neutrophil migration pathways, and impaired cellular metabolism Fig. S3B. Aged Neu2 cells also exhibited an enhanced inflammation signature, increased levels of genes regulating myeloid leukocyte differentiation activation, and downregulated genes regulating metabolism Fig. S3D. Taken together, these results show that aged wounds are largely populated with neutrophils with reduced capacity to stimulate wound resolution and protect against tissue damage.

### Diminished functionality of macrophages in aged compared to young mice

**Fig. 6:**
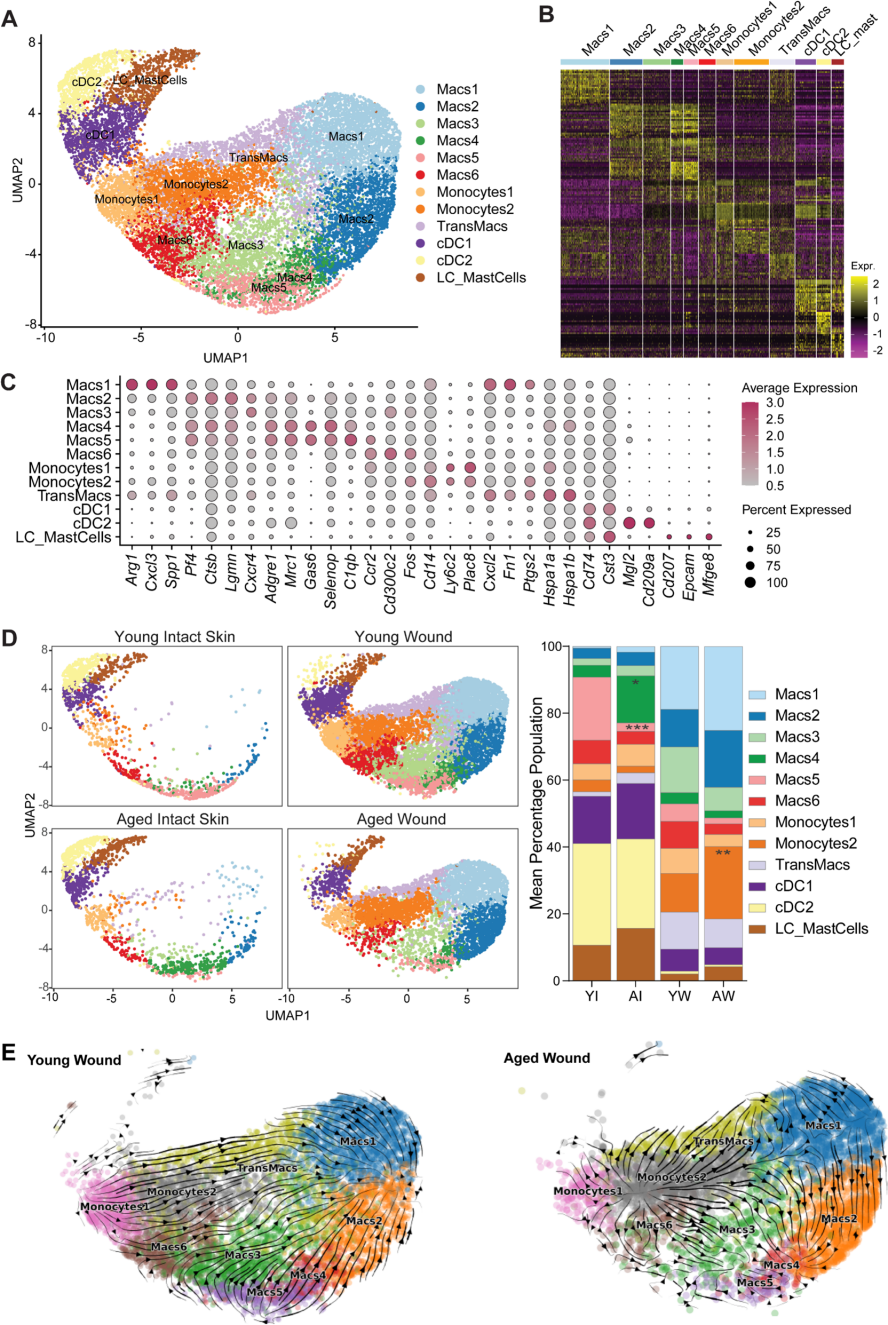
Aged skin tissue-resident macrophages exhibit altered functional pathways in comparison to young ones.** A** UMAP visualisation of macrophages and dendritic cell subtypes in young and aged mouse skin and wounds. **B** Heatmap of top 20 differentially expressed genes in each macrophage subpopulation. **C** Dot plot showing the expression of marker genes for each identified subpopulation. **D** (**left**) UMAP shows the distribution of macrophage-dendritic subpopulations in young and aged skin and Day 3 wounds. (**right**) Bar chart shows the mean proportional distribution of all subpopulations in young and aged intact and wounded tissues. *n* = 3–4 mice per group. *p < 0.05, **p < 0.001, ***p < 0.001, two-way ANOVA with a Tukey multiple comparisons test. **E** RNA velocity analyses in young and aged wound macrophage populations. *Macs* macrophages*, TransMacs* transitioning macrophages, *cDC* conventional dendritic cells, *LC* Langerhans

We previously performed bulk RNA sequencing on young and aged macrophages from Day 3 wounds that showed specific ageing-related changes to the macrophage transcriptome (Dube et al. 2022a). Here, we sought to further elucidate these changes at the single-cell level. In total, 18,111 cells of monocyte-macrophage lineage were analysed and subclassified into 12 defined subpopulations Fig. 6A–C. We identified 6 macrophage groups, Macs1 (*Arg1*^*hi*^* Cxcl3*^*hi*^), Macs2 (*Ctsb*^*hi*^* Lgmn*^*hi*^), Macs3 (*Cxcr4*^*hi*^), Macs4 (*Mrc1*^*hi*^* Gas6*^*hi*^* Selenop*^*hi*^), Macs5 (*Mrc1*^*hi*^* Gas6*^*hi*^* C1qb*^*hi*^), and Macs6 (*Ccr2*^*hi*^* Cd300c2*^*hi*^* Fos*^*hi*^), based on transcriptomic states Fig. 6A–C***,*** Supplementary File 6. We also identified two monocyte subclusters, Monocyte1 and Monocyte2, expressing high levels of *Ly6c2*, *Cd14* and *Plac8* but differential levels of *Lyz2*. Macrophage-monocyte subpopulations likely exist on a continuum, and some may represent differentiating transitioning states. We noted a population between monocytes and macrophages and termed it transitioning macrophages (TransMacs, *Hspa1a*^*hi*^* Hspa1b*^*hi*^). Dendritic cell populations were divided into *Cd74*^*hi*^* Cts3*^*hi*^ cDC1 and *Cd74*^*hi*^* Cd209a*^*hi*^* Mgl2*^*hi*^ cDC2. Another cluster contained *Cd207*^*hi*^* Epcam*^*hi*^ Langerhans cells as well as *Mfge8*^*hi*^ Mast cells (LC_MastCells) Fig. 6A–C.

The cellular composition in skin and wounds underscored heterogeneity in macrophage populations. Macs4 and Macs5 cells were highly distributed in skin and not in wounds. Macs1, Macs2, Mac3 and all monocyte populations were associated with wound healing. Macs6 cells were equally distributed in both intact and wounded tissues Fig. 6D. Interestingly, all cell types were differentially distributed in young versus aged tissues, indicating significant ageing-related alterations in the molecular differentiation of these cells, especially in response to wounding. To investigate this, we performed RNA velocity analysis on macrophage and monocyte populations from wounds to infer the differentiation trajectories of these cells. This computational method analyses the amount of spliced and unspliced RNA transcripts to estimate the direction and speed of gene expression change within each cell, thereby allowing prediction of future transcriptional states of cells in a given single-cell dataset (Bergen et al. 2020). In young wounds, RNA velocity streams revealed two putative terminal cell states, Macs1 and Macs2. Monocytes1 streamed towards the Monocytes2 and TransMacs populations which ultimately gave rise to Macs1 cells. Macs2 originated from the skin-resident-like population Macs5 transitioning through Macs3 Fig. 6E, C. By contrast, we found major disruptions in the transformation of monocytes to macrophages in aged wounds, such that velocity flows of aged wounds did not clearly delineate any terminal cell states or differentiation trajectories Fig. 6E.

This finding was further supported by the altered expression of key transcription factors regulating monocyte-to-macrophage transition, such as *Fos*, *Jun*, *Nr4a1*, *Klf4* and *Stat1* in aged wound monocyte subpopulations compared to young Fig S4A. We validated the reduced expression of these key factors in bulk RNA-sequencing data from macrophages isolated from young and aged day 3 wounds Fig. S4B, data from Dube et al. (Dube et al. 2022a). Interestingly, *Fos*, *Jun* and *Nr4a1* are direct targets of Cebpa and Spi1 (Diamant et al. 2024), two cooperative regulators of macrophage differentiation (Pundhir et al. 2018; Yeamans et al. 2007). Cebpa drives Spi1 expression and protein function, so we analysed their gene expression levels and Cebpa protein localisation in day 3 wounds of young and aged mice. We found reductions in gene expression of these transcription factors and a significantly higher percentage of macrophages with cytoplasmic localisation of Cebpa in aged wounds compared to young Fig S4C, D.

**Fig. 7:**
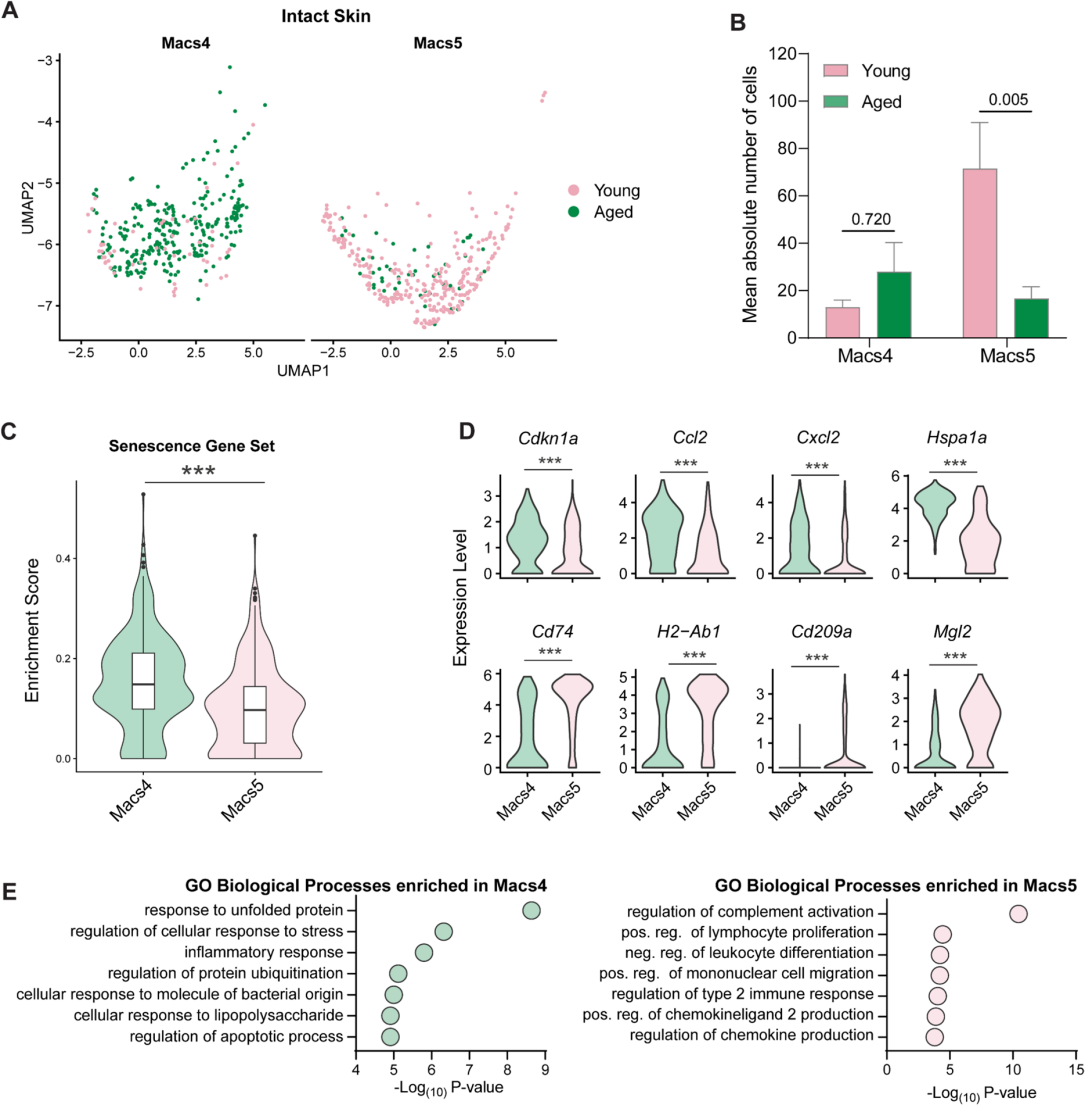
Two functionally defined types of skin-resident macrophages are differentially associated with young (Macs5) and aged (Macs4) skin.** A** UMAP visualisation of the distribution of macrophage populations Macs4 and Macs5 in young and aged intact skin. **B** Bar chart of the absolute number of Macs4 and Macs5 macrophages in young and aged intact skin. Two-way ANOVA with a Bonferroni multiple comparisons test (*n* = 3–4 mice per group, Data are presented as mean ± SEM). **C** Violin plots displaying enrichment score for the SenMayo senescence geneset in intact skin Macs4 and Macs5 populations (***p < 0.001, Two-tailed unpaired *T*-test)**. D** Violin plots showing differences in gene expression levels of *Cdkn1a, Ccl2, Cxcl2, Hspa1a, Cd74, H2-Ab1, Cd209a, Mgl2* genes in Macs4 and Macs5 subpopulations in young and aged intact skin (***p < 0.001, Two-tailed unpaired *T*-test). **E** GO analyses illustrating enriched biological processes in Macs and Macs5 cells from young and aged intact skin. *Macs* macrophages, *GO* gene ontology*, pos* positive, *neg* negative, *reg* regulation

To further investigate what is driving impaired monocyte-macrophage transitioning pathways in the aged wounded tissues, we reinspected intact skin macrophages to check for intrinsic differences. We noted that the skin-resident macrophage population, Macs5, which comprised 19% of the total macrophage population in intact young skin and gave rise to Macs2 and Macs3 in the young wounds, was significantly diminished in aged skin Fig. 6D, Fig. 7A–B. Instead, aged skin was populated by a larger percentage (15%) of Macs4 cells Fig. 6D, Fig. 7A-B. While both Macs4 and Macs5 expressed high levels of canonical skin macrophage markers such as *Adgre1*, *Mrc1*, *Gas6* and *Pf4* Fig. 6C, *Geneset* scoring revealed that Macs4 cells had significantly higher enrichment scores for SASP-related genes Fig. 7C. Differential expression analyses between Macs4 and Macs5 in the skin further revealed that the expression of genes associated with key macrophage functions was altered in Macs4. Macs4 expressed higher levels of senescence-related gene, *Cdkn1a*, stress-related gene *Hspa1a* as well as pro-inflammatory genes *Ccl2* and *Cxcl2* Fig. 7D, Supplementary File 6. Additionally, Macs4 exhibited a decrease of antigen-presenting associated genes, *H2-Ab1*, *Cd74* and *Mgl2*, and the pathogen-recognition-related gene, *Cd209d*. GO analyses also showed that unlike Macs5, which expressed genes associated with primary macrophage function such as regulation of chemokine production and regulation of complement activation, Macs4 cells were enriched in genes associated with the inflammatory response and regulation of cellular stress response Fig. 7E. Altogether, our data indicate that aged skin tissues are intrinsically populated by senescent, inflammatory Macs4 macrophages, which, unlike Macs5 cells, are unable to support normal monocyte-macrophage transitioning trajectories, resulting in impaired responses to injury.

#### Altered intercellular signalling between resident and infiltrating cell populations during early healing responses in aged wounds

**Fig. 8:**
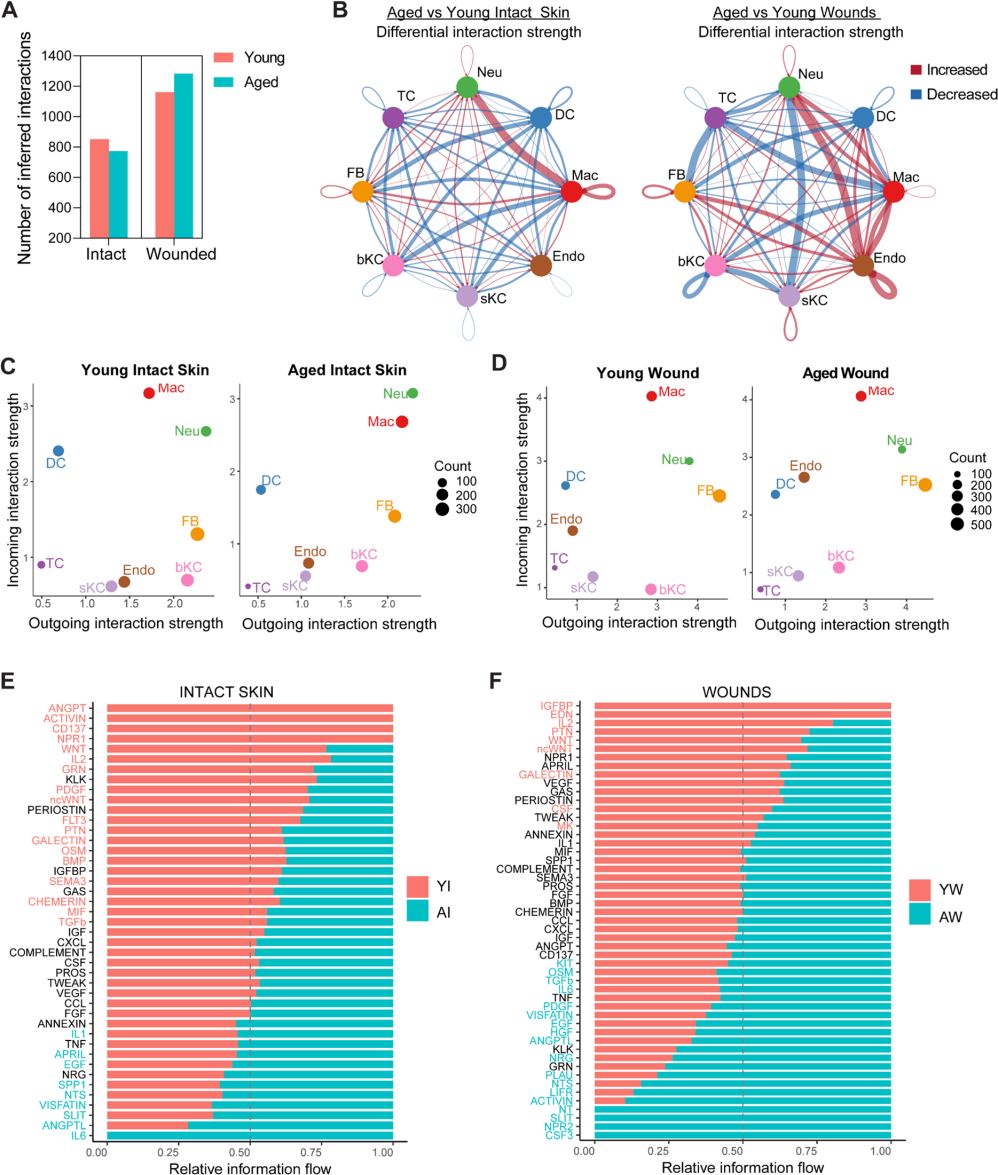
Altered intercellular signalling in the aged skin microenvironment persists during early healing responses.** A** Bar plot showing the number of ligand-receptor interactions inferred in young and aged intact skin and wounds. **B** Circle plot illustrating differential interaction strength between aged and young intact skin (left) and aged and young wounds (right). **C–D.** Scatter plot showing total incoming and outgoing interaction strengths for each cell type in young and aged skin and wounds. Dot size corresponds to the number of interactions in each cell population. **E–F** Bar plot illustrating relative signalling pathway information flow in young and aged intact skin and wounds. *Neu* neutrophils, *DC* dendritic cells*, Mac* macrophages, *Endo* endothelial cells, *sKC* suprabasal keratinocytes, *bKC* basal keratinocytes, *FB* fibroblasts, *TC* T cells. *YI* young intact skin, *AI* aged intact skin, *YW* young wound (day 3), *AW* aged wound (day 3)

So far, our data has demonstrated transcriptomic differences in young and aged cells in the skin as well as during the early responses to injury. To assess how changes in the transcriptome of these cells affect the skin and wound microenvironment, we compared cell communication patterns between young and aged skin and wounds using *CellChat* (Jin et al. 2021). We found that the number of inferred ligand-receptor interactions in the skin was reduced in aged compared to young mice. However, there were more inferred interactions in aged wounds than in young, demonstrating altered signalling in the aged Fig. 8A.

We compared the interaction strength between major cell types in young and aged skin and wounds. In intact skin, interaction strengths were generally lower in most cell types in the aged skin compared to the young Fig. 8B. Notably, macrophages in the aged skin exhibited markedly increased outgoing interaction strengths with all other cell types, particularly neutrophils and FBs but weaker incoming interactions compared to young skin. This indicated aberrant signalling in aged skin, with macrophages playing a central role. In aged wounds, we observed stronger interactions between endothelial cells and other cell types involved in the healing progression such as macrophages, neutrophils, and FBs indicative of altered signalling during early healing phases. In addition, macrophages and neutrophils also had enhanced cell communication in the aged wounds indicating that the inflammatory response is heightened compared to young wounds Fig. 8B. Further analyses revealed that macrophages are dominant senders and receivers of communication signals in both skin and wounds, and their cell signalling landscape is significantly altered in response to both ageing and injury Fig. 8C–D, Fig S5.

Next, we assessed if pathways altered in aged skin were also similarly changed in aged wounds. Our results revealed a shift in signalling pathways in response to ageing. Several signalling pathways, including WNT, IL2, PTN (pleiotrophin) and Galectin pathways, which regulate tissue homeostasis and healing, had significantly reduced information flow in both aged skin and wounds Fig. 8E–F. In contrast, IL6 and ANGPTL (angiopoietin-like) signalling pathways previously implicated in ageing and ageing-related disease had increased ligand-receptor interactions in aged skin as well as wounds Fig. 8E–F (Maggio et al. 2006; Morinaga et al. 2018). Altogether, this data demonstrates that signalling in the skin microenvironment is altered by ageing and these ageing-related changes persist following injury and ultimately impact wound healing.

## Discussion

Progression of wound healing is delayed in aged mammals and previous studies implicate an impaired inflammatory response suggestive of altered early healing responses to injury in these tissues (Ashcroft et al. 1998; Dube et al. 2022a; Swift et al. 2001; Vu et al. 2022). Nonetheless, a careful characterisation of cumulative changes in skin cell subsets that contribute to impaired early responses to wounding in aged tissues is still lacking. In the present study, we performed single-cell RNA sequencing on young and aged intact mouse skin and wounded tissues 3-days post-injury to investigate specific cell-intrinsic changes that impact early responses to injury, leading to poorer healing outcomes in aged wounds. Murine wound healing differs from human in some aspects, such as the dependence on wound contraction in murine skin, but major wound healing cell types, such as those detailed in this study, and processes such as inflammation, angiogeneiss and proliferation, and extra cellular remodelling are consistent between mammalian species. Furthermore, wound response genes are highly conserved (Khalid et al. 2022; Wang et al. 2024). Our study focused on major cell types including keratinocytes, fibroblasts, macrophages and neutrophils in young and aged skin and wounds, all of which are relevant to human wound healing. Under homeostasis, young and aged skin tissues exhibited comparable cell type compositions and relative cellular distributions, consistent with findings from recent studies in mouse and human skin (Dube et al. 2022a; Ge et al. 2020; Vu et al. 2022; Zou et al. 2021).

We found that ageing had a significant impact on cell distribution as well as transcriptomic states at the subpopulation level. Aged tissues had fewer proliferating *Mki67*^hi^ KCs, which exhibited downregulated cell cycle and cell division genes, consistent with previous observations in the mouse and human skin (Dube et al. 2022b; Rübe et al. 2021). Aged KCs also exhibited impaired differentiation trajectories, altered proportional distributions of subpopulations driven by reduced expression of differentiation transcription factors (*Gata3*, *Bhlhe40),* and increased levels of senescence genes (*Cdkn1a*, *Ccnb1ip1*), particularly in the basal progenitor populations (Cyc1). Age-related induction of senescence and reduced proliferative capacity in KCs have been previously described (Dreesen et al. 2013; Ge et al. 2020; Rübe et al. 2021; Wang et al. 2017). Our data adds to this by demonstrating that impaired function and differentiation of proliferative basal cells results in a reduction of terminally differentiated cells which in turn compromises barrier function in aged skin.

We recently demonstrated that F4/80^+^ CD64^+^ macrophages were reduced in aged skin (Dube et al. 2022a). In this study, we also found fewer total resident macrophage populations in aged wounds and defined two major resident macrophage cell populations differentially associated with young (Macs5) and aged (Macs4) skin. Aged skin-associated population Macs4 had increased expression of stress response, inflammation, apoptosis and senescence-associated genes. In support of this finding, a recent study also demonstrated that aged macrophages in the human skin had a heightened proinflammatory signature. However, the study deduced that the inflammatory state of macrophages in aged skin was a response to senescent-aged FBs in the microenvironment (Gather et al. 2022). Conversely, we show evidence of senescent macrophages and FBs expressing high levels of *Cdkn1a* as well as other SASP-related factors in aged skin. It remains to be determined whether senescent macrophages are spatially linked to senescent aged FBs and how their interactions influence inflammation in aged skin. Senescence is also a potential target of impaired wound healing in human patients as well, with prolonged senescence being linked to chronic wound development (Wilkinson & Hardman 2020).

Notably, in aged wounds, we found that skin tissue resident-to-wound macrophage transitioning was defective, likely due to the loss of the resident-like Macs5 population, which is replaced by Macs4 cells exhibiting senescence and inflammatory phenotypes. Further, disrupted monocyte-macrophage transitioning may also be exacerbated by the loss of transcription factors such as *Nr4a1, Jun* and *Fos* in the monocyte populations, which are required for this transition (Coccia et al. 1999; Hilgendorf et al. 2014; Valledor et al. 1998). A recent study also demonstrated disordered monocyte-macrophage transitioning patterns in aged macrophage populations from Day 4 wounds. Interestingly, they observed that in aged wounds, monocyte-macrophage transitioning patterns at later healing time points (Day 7) were restored and appeared similar to those seen on Day 4 in young wounds suggestive of a delay in the establishment of transitioning patterns in aged wounds (Vu et al. 2022). These findings further support that early healing responses are significantly impacted by ageing following injury.

In summary, our data define an accumulation of transcriptomic changes in aged resident skin cell populations which impair function and result in globally altered intercellular signalling in the aged skin microenvironment. We show that impaired signalling in the aged tissues persists after an injury and alters early wound responses and poor healing progression. Our work highlights a cumulative contribution of functionally defective fibroblasts, neutrophils and macrophages to poor healing in aged mice. However, the influence of ageing on the spatial location of all these cell populations remains to be determined. Future studies utilising spatial RNA sequencing will be useful in validating how ageing influences cellular spatial distribution in early wound healing responses, so that impaired cell populations can be precisely targeted to improve healing. Moreover, as not all transcriptional changes are reflected by equivalent changes in protein products, future studies should include targeted protein analyses in both mouse and human. Further in vivo and in vitro functional validation studies are also required to confirm the mechanisms regulating impaired cellular function in aged skin and wounds.

## Methods

### Mice

Female C57BL/6 J wild-type young (3 months) and aged (22–24 months) mice were bred and housed at the A*STAR Biological Resource Centre (*Singapore)*. All mice were maintained under a temperature and humidity-controlled environment with a 12-h light–dark cycle and free access to food, water and environmental enrichment devices. All protocols involving animals were approved by the Agency for Science, Technology and Research Institutional Animal Care and Use Committee, Singapore (A*STAR-IACUC, Reference number: 191495). All experiments were performed according to the ethical committee’s guidelines and regulations. The number of animals used in each study was determined through a power analysis. A minimum of 3 animals was used in each group.

### Wounding surgery

Female mice (aged, n = 3 mice, young, n = 4 mice) were placed in induction chambers and anaesthesia was induced using 2% isoflurane delivered in 100% oxygen until toe pinch reflexes were inhibited. Back skin was shaved with an electric razor and further depilated using hair removal cream. All mice were wounded during the telogen phase of the hair cycle phase to avoid cycle-specific effects. Skin was disinfected using povidone-iodine swabs and 0.1 mg/kg buprenorphine was administered via subcutaneous injection for post-surgical pain relief. Two circular 10 mm diameter impressions were then outlined on the dorsum, on each side of the midline and full-thickness wounds were cut using 100 mm iris curved scissors (*World Precision Instruments*). Mice were allowed to recover and monitored daily until the end of the experiment.

### Tissue processing and single-cell isolation

On day 3 post-wounding, mice were sacrificed using carbon dioxide, and wounds were excised, including a 2-mm margin. Distal intact back skin was also excised as a representation of normal unwounded skin. Prior to digestion, the fat tissue layer was gently scraped, and tissues were cut into small pieces using a scalpel. Tissues were transferred into C-tubes and agitated at 37 °C for 90 min in complete RPMI media containing 0.25 mg/ml Liberase TL (*Roche*) and 0.1 mg/ml DNase 1 (*Sigma*). Tissues were dissociated using gentleMACS dissociator ‘m_muscle_01’ protocol (*Miltenyi*). The single cell suspension was passed through a 100 µm cell strainer, washed 3X with PBS containing 3% FBS and 5 mM EDTA, and passed through a 40 µm cell strainer. To isolate live single cells, 3 µm DAPI was added to the suspension and incubated for 10 min in the dark to exclude dead or dying cells. Live single cells were sorted using a BD Influx cell system. All sorted samples had a viability of greater than 75% and cells were processed within 30 min of sorting.

### Single cell rna sequencing

For single-cell RNA experiments, isolated cells were resuspended in 0.04% BSA in PBS at a concentration of 800 to 1000 cells/µl. Single-cell experiments were performed using Chromium Next GEM Automated Single Cell 3ʹ cDNA Kit v3.1 on the 10X Genomics Chromium Connect platform (*10X Genomics*) according to the manufacturer’s instructions. 10 µl of each sample (targeted recovery of 5000 cells per sample) was added into the supplied 96-well skirted plate (maximum capacity of 8 samples per run) and placed into the machine and single-cell libraries were generated using the system’s automated workflow. A total of 23 libraries from young and aged intact skin and wounds were created and sequenced on the NovaSeq 6000 Illumina platform to an average of 114,000 reads per cell. A summary of all the samples processed from 3 aged and 4 young mice is shown in Table S2.

### Initial single-cell quality control

Raw base call files were demultiplexed and aligned to the mouse genome reference mm10 using 10 × Genomics Cell Ranger v4.0.0. The read counts for all samples were uploaded and log-normalised in Seurat v4 in R v4.2.0 (Hao et al. 2021; R_Core_Team 2021). As a first QC step, we kept barcodes with 300–7500 detected genes, 500–75,000 unique molecular identifiers and a percentage of mitochondrial reads below 15%. Next, we identified intra-sample heterotypic cell type doublets from each library, assuming a 1% doublet formation rate per 1000 recovered cells, using DoubletFinder v2.0 (McGinnis et al. 2019). We then assessed for batch effects visually and noted that the major separation in the dataset was due to biological factors rather than technical factors (Fig S1C). After a thorough assessment, we concluded that further batch correction introduced artefacts and obscured biological signals. As such, batch correction was not applied.

### Major cell type identification

The remaining 92,934 singlets across all libraries were clustered using the reference component analysis v2 (RCA2) with graph-based clustering with resolution 0.1 using GlobalPanel reference (Li et al. 2017). The gene symbol in GlobalPanel was converted from human to homologous mouse gene symbols using non-ambiguous mapping in *biomaRt* (Durinck et al. 2009). We identified 8 major populations in the young and aged skin and wounds, empirically via FindAllMarkers(), and by examining the published canonical markers. Gene expression markers were visualised using Nebulosa (Alquicira-Hernandez & Powell 2021).

### Major cell type quality control, analysis and fine clustering

Each major cell type was analysed independently as follows. First, we performed cell type-specific QC (ctQC) by setting thresholds on the distribution of the number of detected genes, the number of unique molecular identifiers and the percentage of mitochondrial reads (Lakshmanan et al. 2024). Next, the data were log-normalised and highly variable genes were identified using *DUBStepR* (Ranjan et al. 2021). The data was scaled before running linear dimensionality reduction using Principal Component Analysis. The optimal number of principal components was determined using the ElbowPlot() function. The 20-nearest neighbourhood graph and shared nearest neighbourhood graph were constructed. Fine clusters were identified using the Louvain community detection algorithm in Seurat using the appropriate resolution and published canonical markers. For comparisons between young and aged groups, cell populations across different conditions were presented as percentages rather than absolute numbers. This approach was taken because the total number of cells obtained from each sample varies due to dissociation and sequencing efficiency. Presenting the data as percentages normalises these differences and allows for unbiased comparisons across conditions. This way, the results reflect biological differences within the subpopulations rather than technical differences.

### Differential expression analysis

Differentially expressed genes (DEGs) were extracted using DESeq2 within the *FindMarkers* function in Seurat, following aggregation of gene expression levels by sample. DEGs were visualised using R packages *EnhancedVolcano* (Blighe et al. 2024). Gene ontology analyses were conducted using the *DAVID* and *EnrichR* online resources, and Gene set enrichment analyses were performed using *clusterProfiler* (Dennis et al. 2003; Ranjan et al. 2021; Wu et al. 2021; Xie et al. 2021). Senescence gene set scoring was performed using the SenMayo geneset curated by Saul et al. (Saul et al. 2022).

### Trajectory analyses using monocle3

Pseudotime trajectory inference on keratinocytes was performed using the R package *Monocle3* (Cao et al. 2019). Briefly, a *cell_data_set* class object containing keratinocytes from young and aged intact skin was created from *Seurat*. Single-cell trajectories were computationally constructed using the *learn_graph* and *order_cells* functions for both young and aged keratinocytes with Cyc1 keratinocytes set as the root population. Differential expression analysis was then performed to identify genes and transcription factors that were changed as a function of pseudotime using graph autocorrelation analysis (to find genes that vary between clusters over a trajectory) or regression analysis (to assess if changes in genes over a trajectory are influenced by age).

### RNA velocity

RNA velocity analyses were performed using *scVelo* (Bergen et al. 2020). First, we created loom files of spliced and unspliced counts from the BAM files for all samples using the Python package *Velocyto* (La Manno et al. 2018). The spliced and unspliced data matrices were then merged with gene expression count matrices, creating an *Anndata* object. RNA velocities were computed and visualised using dynamical modelling for young and aged wound macrophages to identify differences in monocyte-macrophage transitioning.

### Cell chat

Cell-to-cell communication changes were inferred using *CellChat* and *CellChatDB* (Jin et al. 2021). Briefly, we created separate *Cellchat* objects for young and aged wounds and computed cell communication networks using the ‘secreted signalling’ subset of the *Cellchat* database. Inferred networks were merged for differential analysis and to explore altered signalling pathways in aged skin and wounds.

### Comparison with publicly available data

To validate changes observed in macrophages, bulk RNA sequencing raw counts of macrophages sorted from young and aged Day 3 wounds were obtained from Dube et al. (Dube et al., 2022a). Gene expression counts for *Fos*, *Jun*, *Stat1*, *Nr4a1* and *Klf4* were normalised to the housekeeping gene *H2ac18*.

### Immunofluorescence staining

5 µm young and aged skin and wounded tissue sections were cut from formalin-fixed paraffin-embedded (FFPE) blocks. Sections were deparaffinised in xylene and rehydrated in decreasing concentrations of ethanol. Sections were rinsed in water, and antigen retrieval was performed in a 2100 Antigen Retriever (*Aptum Biologics*) using the IHC Antigen Retrieval Solution at pH 6 or pH 9 (*Invitrogen*). Non-specific binding was blocked using 1% bovine serum albumin and 25% normal goat serum. Sections were then incubated with 100μL of the following primary antibodies overnight at 4 °C: mouse anti-Collagen, Type I pro-peptide (2 µg/ml, SP1.D8, *DHSB*), rabbit anti-Keratin 14 (1 in 200, Poly19053, *Biolegend*) or rabbit anti-CEBPA (1 in 100, sc-61X, *Santa Cruz Biotechnology*) and rat anti- F4/80 (1:100, ab6640, *Abcam*). Sections were washed with PBS + 0.1% Tween (PBST) and incubated with goat anti-mouse Alexa Fluor 488, goat anti-rabbit Alexa Fluor 594, donkey anti-rabbit Alexa Fluor 555, or donkey anti-rat Alexa Fluor 488 secondary antibodies (1 in 400, *Invitrogen*) for 1 h at room temperature in the dark. Sections were counterstained with DAPI for 15 min. Sections were washed with PBST and mounted with Prolong Gold antifade mountant (*Invitrogen*). Tissues were imaged with a Nikon Eclipse Ti confocal microscope and associated software, NIS-ElementsAR. Four fields of view were imaged per tissue section at a resolution of 1024 × 1024 pixels using a 20X or 40X lens. All images were visualised and quantified using ImageJ software. Researchers were blinded during imaging and analyses, and samples were decoded after analyses were completed.

### Statistical analyses

Statistical analyses were performed on *GraphPad Prism 9* and *R*. Two-way ANOVA tests with Tukey multiple comparisons were used to compare differences between groups. Unpaired t-tests were used to compare between two groups. Statistical significance is set at p < 0.05 unless stated otherwise.

## Supplementary Information

Below is the link to the electronic supplementary material.

Supplementary file1 (PDF 1769 KB)

Supplementary file2 (XLSX 267 KB)

Supplementary file3 (XLSX 524 KB)

Supplementary file4 (XLSX 1082 KB)

Supplementary file5 (XLSX 1422 KB)

Supplementary file6 (XLSX 540 KB)

## Data Availability

The datasets generated and analysed during the current study have been deposited in the GEO database, accession number: GSE267091.

## Code availability

No custom code was generated in this study. The software packages used are publicly available and acknowledged in the relevant sections of the methods.
