# Supplementary material for: Intrinsic changes in cell differentiation and identity drive impaired wound healing in aged female murine skin
Source: Biogerontology. 2025 Nov 1;26(6):200. doi: 10.1007/s10522-025-10340-w (PMC12579693; doi:10.1007/s10522-025-10340-w)
Supplement: Supplementary file 1 — Supplementary file1 (PDF 1769 KB) [file 10522_2025_10340_MOESM1_ESM.pdf]

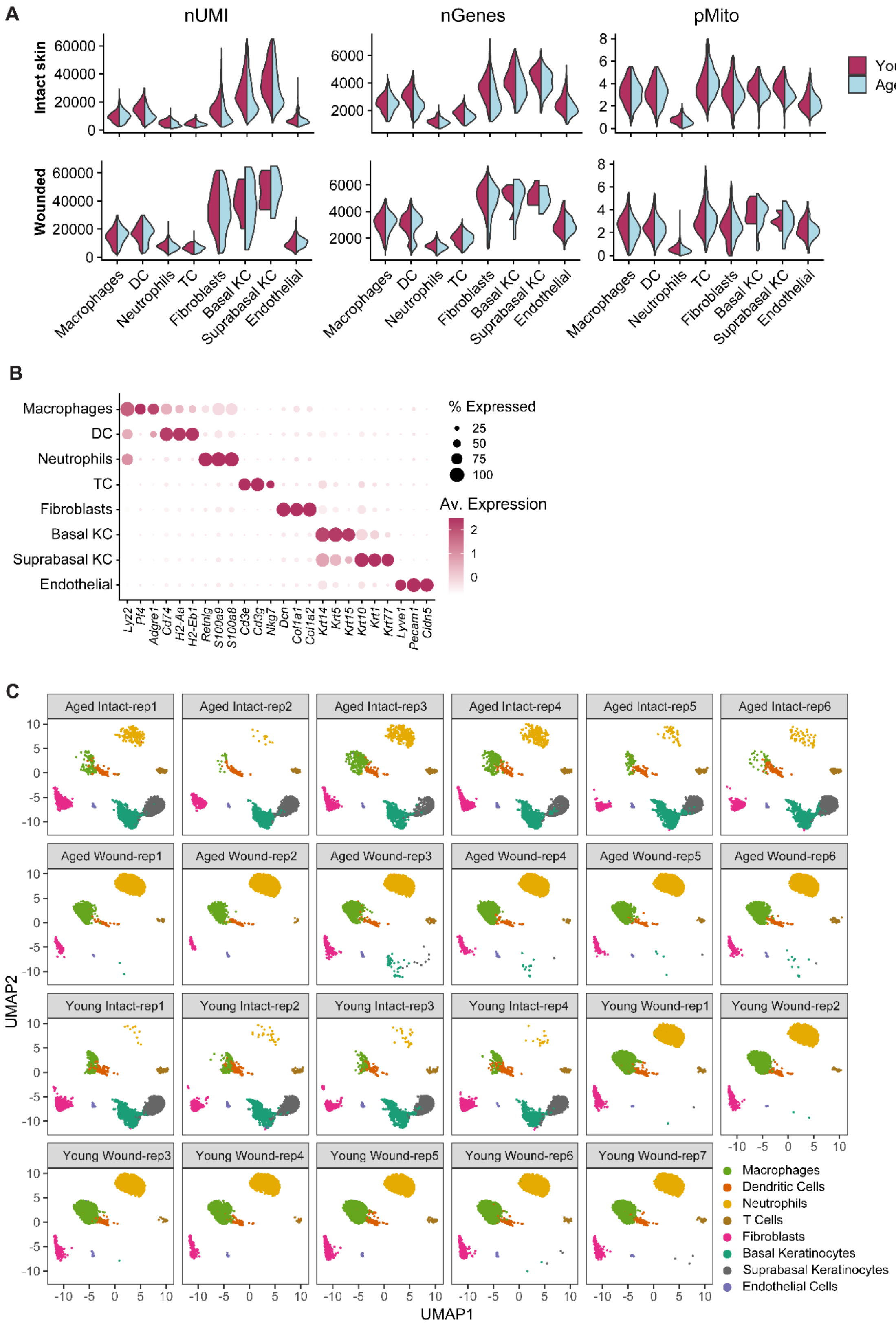

Supplementary Figure S1. Single-cell RNA sequencing quality control. (A) Post quality control distribution of the number of unique molecular identifiers (nUMI), number of genes (nGenes) and percentage mitochondrial reads (pMito) in young and aged intact skin and

wounded cell populations. (**B)** Dot plot showing average expression of top 3 canonical marker genes of each identified major cell type. **(C)** UMAP visualisation of the distribution of all cell types in each replicate sample.

Supplementary Table S1: List of samples with the GEO accession ID and number of cells after quality control.

		GEO Accession ID		Number of cells after QC	
**Group**	**Animal ID**	**Biopsy 1**	**Biopsy 2**	**Biopsy 1**	**Biopsy 2**
Aged Intact Skin	AI-M2	GSM8259838	GSM8259841	4283	3447
	AI-M4	GSM8259836	GSM8259839	2641	3388
	AI-M6	GSM8259837	GSM8259840	3304	4591
Aged Day 3 Wounds	AW-M2	GSM8259844	GSM8259847	2674	4003
	AW-M4	GSM8259842	GSM8259845	3345	3218
	AW-M6	GSM8259843	GSM8259846	2760	3739
Young Intact Skin	YI-M1	GSM8259851	*not collected*	4329	.
	YI-M3	GSM8259852	*not collected*	2672	.
	YI-M5	GSM8259850	*not collected*	2954	.
	YI-P1	GSM8259848	*not collected*	3014	.
Young Day 3 wounds	YW-M1	GSM8259853	GSM8259858	3637	3501
	YW-M3	GSM8259855	GSM8259856	3016	3003
	YW-M5	GSM8259854	GSM8259857	3721	3707
	YW-P1	GSM8259849	*not collected*	3723	.

Multiple biopsy samples were taken from aged intact, aged wounded and young wounded animals to increase the number of cells recovered. The total number of cells per group: 21654 (Aged Intact), 19739 (Aged Wounded), 12969 (Young Intact) and 24308 (Young Wounded).

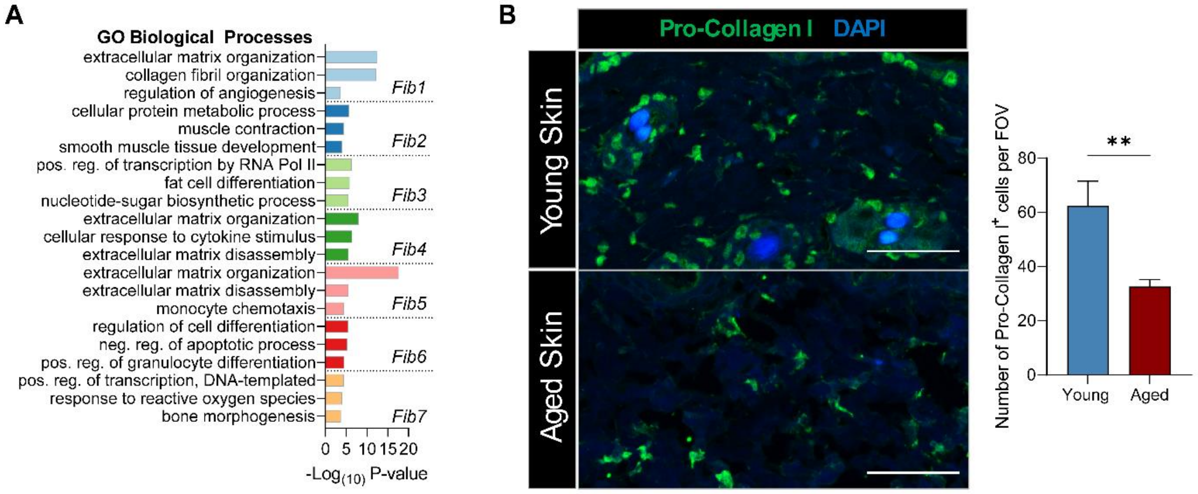

Supplementary Figure S2. (A) Enriched GO terms (biological processes) in defined fibroblast clusters in young and aged skin. **(B)** Representative confocal images of Collagen, Type I pro-peptide (pro-collagen Type I or Pro-Collagen I) staining in young and aged mouse dermal skin. Scale bar =50μm. Bar chart shows the number of Pro-Collagen I^{+} cells per FOV in young and aged skin (n=4, **p<0.01, Two-tailed unpaired T-test)**.**

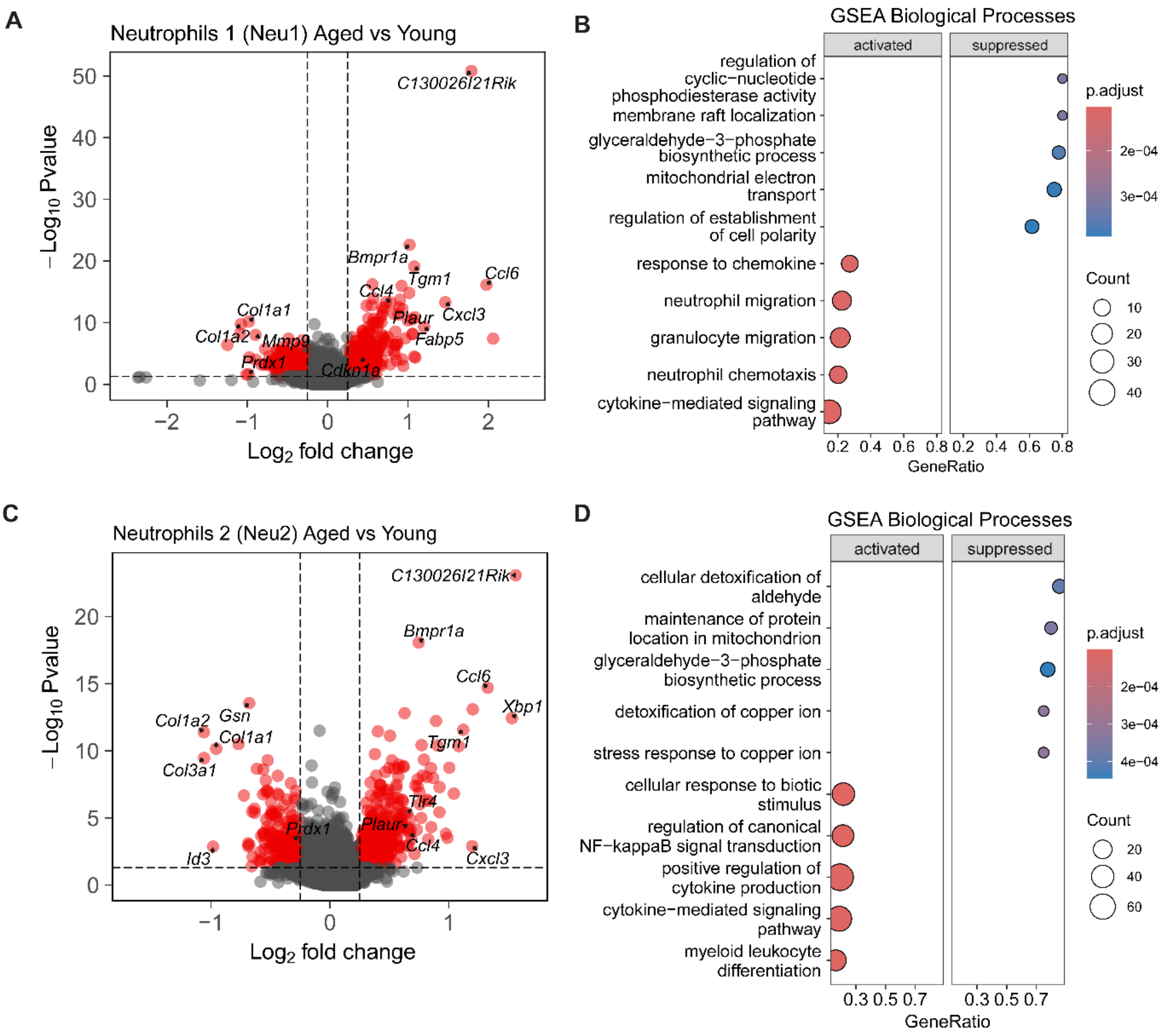

Supplementary Figure S3. Pathway enrichment analyses in young and aged wound neutrophils populations. (A&C) Volcano plots showing differentially expressed genes in aged wounds Neu1 and Neu2 cell populations in comparison to young wounds (adjusted_pvalue<0.05, log2_foldchange>0.25, data analysed using DESeq2). **(B&D)** Gene set enrichment analyses of up and downregulated biological processes in Neu1 and Neu2 cells in aged versus young wounded tissues.

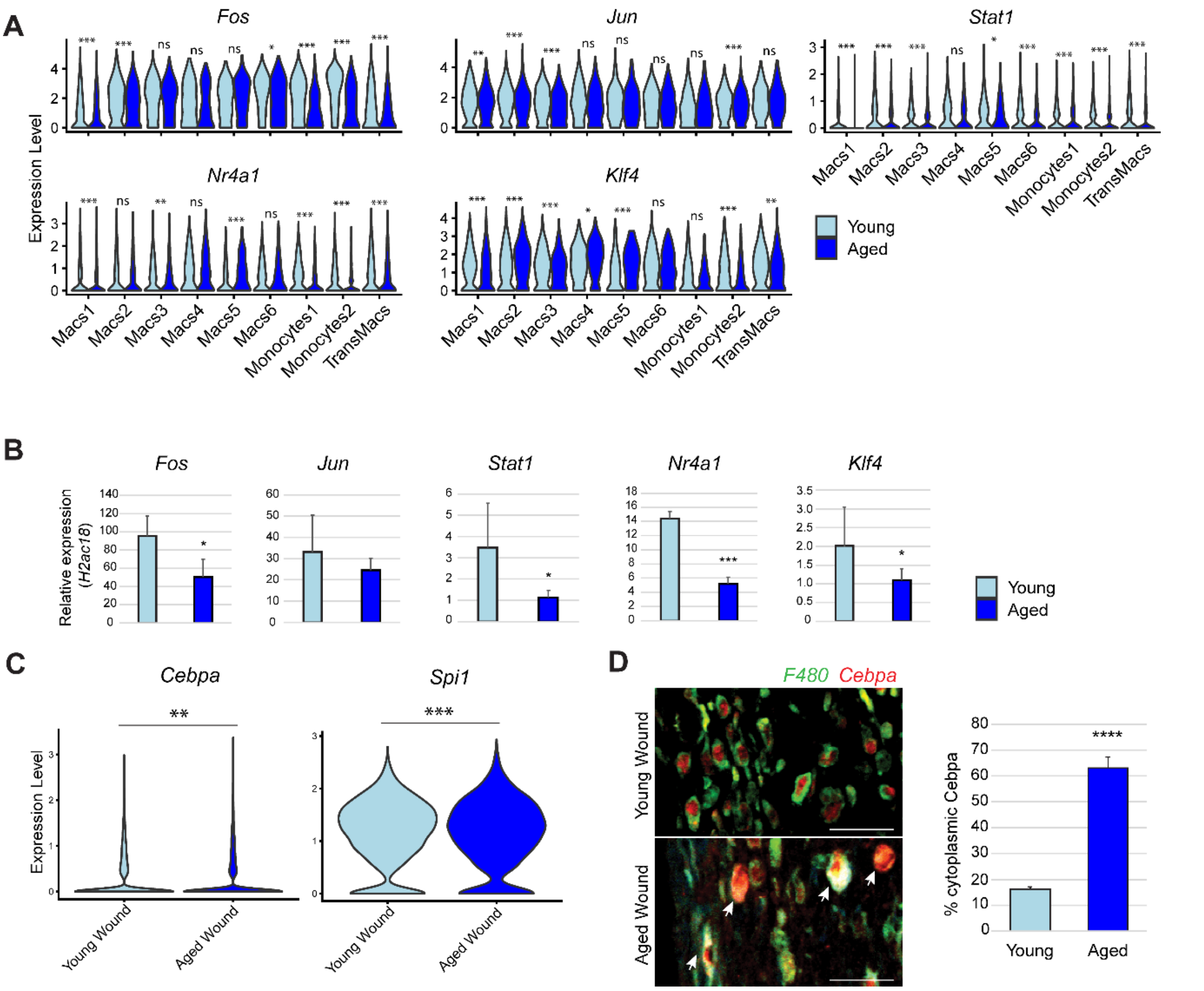

Supplementary Figure S4. Key transcription factors that regulate monocyte-to-macrophage transition are downregulated in the monocytes from aged wounds. (A) Violin plots showing differences in gene expression levels of *Fos*, *Jun*, *Stat1*, *Nr4a1,* and *Klf4* genes in young and aged monocyte-macrophage subpopulations in Day 3 wounds (*p<0.05, **p<0.01, ***p<0.001, ns=not significant, Two-tailed unpaired T-test). **(B)** Relative expression of *Fos*, *Jun*, *Stat1*, *Nr4a1,* and *Klf4* transcripts to *Hist2ac18* from RNA-seq of F4/80^{+} macrophages derived from Day 3 wounds (n=3, *p ≤ 0.1, ***p ≤ 0.01, two-tailed unpaired T-test). **(C)** Violin plots showing differences in gene expression levels of *Cebpa* and *Spi1* genes in young and aged macrophage subpopulations (combined) in Day 3 wounds (**p<0.01, ***p<0.001, Mann-Whitney test). **(D)** Representative confocal images of F4/80 and Cebpa protein staining in young and aged mouse Day 3 wounds. Arrows indicate F4/80^{+}

cells with cytoplasmic Cebpa protein localisation. Scale bar =20μm. Bar chart shows the percentage of F4/80$^+$ cells with cytoplasmic Cebpa protein localisation in young and aged wounds (n=4, ****$p \leq 0.001$, two-tailed unpaired T-test).

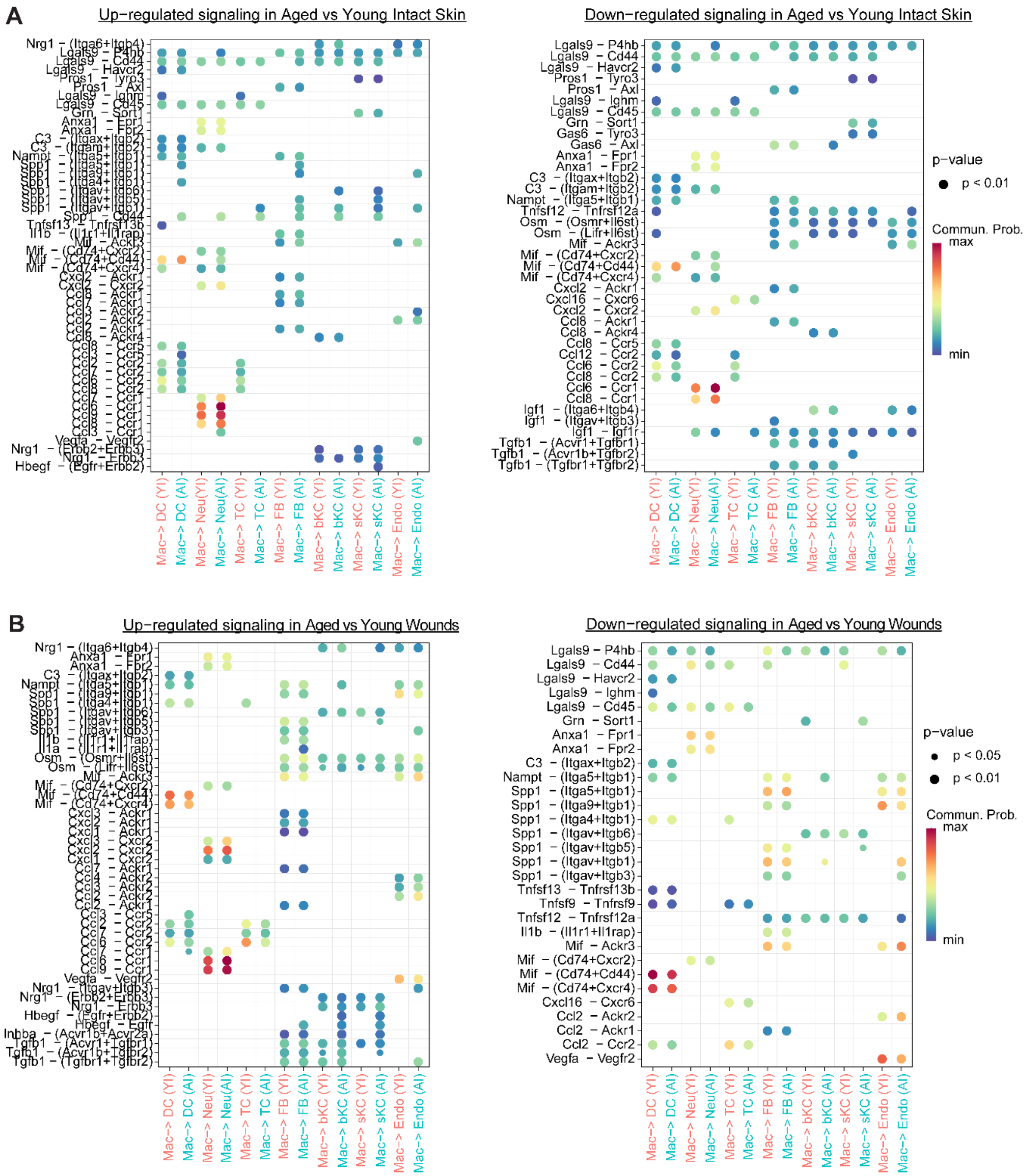

Supplementary Figure S5. Cell-cell communication in Macrophages from young and aged skin and Day 3 wounds. (A-B) Bubble showing upregulated and downregulated ligand-receptor pair interactions between macrophages and other populations in young and aged skin and wounds.

Supplementary Table S2: Summary of Experimental Design and Sequencing Output

Sample	Group	Status	Collection Day	Sorting Order	Cell viability	Sequencing Lane	Estimated Number of Cells	Mean Reads per Cell	Median Genes per Cell
YI_P1	Young	Intact	4	8	88%	5	5,050	91,534	3,250
YI_M1	Young	Intact	1	4	92%	2	6,274	88,589	3,519
YI_M3	Young	Intact	2	5	84%	3	4,608	133,129	3,428
YI_M5	Young	Intact	3	4	85%	4	5,373	96,667	3,219
YW_P1	Young	Wounded	4	8	87%	5	4,738	93,170	1,781
YW_M1_rep1	Young	Wounded	1	1	87%	1	4,548	96,801	1,992
YW_M1_rep2	Young	Wounded	1	5	92%	2	4,592	143,802	1,860
YW_M3_rep1	Young	Wounded	2	3	82%	1	4,037	101,848	1,690
YW_M3_rep2	Young	Wounded	2	4	87%	3	4,069	140,133	2,061
YW_M5_rep1	Young	Wounded	3	1	88%	1	4,705	100,108	2,089
YW_M5_rep2	Young	Wounded	3	6	85%	4	4,741	131,813	1,930
AI_M2_rep1	Aged	Intact	1	3	85%	2	6,305	85,002	2,942
AI_M2_rep2	Aged	Intact	1	6	88%	2	4,925	110,176	2,592
AI_M4_rep1	Aged	Intact	2	1	78%	3	4,480	120,240	2,996
AI_M4_rep2	Aged	Intact	2	7	84%	3	5,719	111,087	3,077
AI_M6_rep1	Aged	Intact	3	2	80%	4	5,781	108,456	2,886
AI_M6_rep2	Aged	Intact	3	5	89%	4	7,055	84,122	2,643
AW_M2_rep1	Aged	Wounded	1	2	83%	1	3,374	152,075	1,401
AW_M2_rep2	Aged	Wounded	1	7	79%	2	4,821	116,920	1,406
AW_M4_rep1	Aged	Wounded	2	2	81%	1	4,409	110,978	1,786
AW_M4_rep2	Aged	Wounded	2	6	86%	3	4,199	142,901	1,750
AW_M6_rep1	Aged	Wounded	3	3	83%	1	3,743	142,859	1,423
AW_M6_rep2	Aged	Wounded	3	7	83%	4	4,570	121,575	1,491

The ARRIVE Essential 10: Compliance Questionnaire

Use this questionnaire to evaluate how well a manuscript complies with the ARRIVE Essential 10. It can be applied to any manuscript describing comparative experiments in living animals, by assessors such as journal staff, editors, or peer reviewers.

Item		Question(s)	Answers
1	Study Design	Are all experimental and control groups clearly identified?	☒ Yes, for at least one experiment<br>☐ No
		Is the experimental unit (e.g. an animal, litter or cage of animals) clearly identified?	☒ Yes, for at least one experiment<br>☐ No
2	Sample Size	Is the exact number of experimental units in each group at the start of the study provided (e.g. in the format '*n*=')?	☒ Yes, for at least one experiment<br>☐ No
		Is the method by which the sample size was chosen explained?	☐ Yes, for at least one experiment<br>☒ No
3	Inclusion & Exclusion Criteria	Are the criteria used for including and excluding animals, experimental units, or data points provided?	☒ Yes, for at least one experiment<br>☐ No
		Are any exclusions of animals, experimental units, or data points reported, or is there a statement indicating that there were no exclusions?	☒ Yes, for at least one analysis<br>☐ No
4	Randomisation	Is the method by which experimental units were allocated to control and treatment groups described?	☒ Yes, for at least one experiment<br>☐ No
5	Blinding	Is it clear whether researchers were aware of, or blinded to, the group allocation at any stage of the experiment or data analysis?	☒ Yes, for at least one experiment<br>☐ No
6	Outcome Measures	For all experimental outcomes presented, are details provided of exactly what parameter was measured?	☒ Yes, for at least one experiment<br>☐ No
7	Statistical Methods	Is the statistical approach used to analyse each outcome detailed?	☒ Yes, for at least one analysis<br>☐ No
		Is there a description of any methods used to assess whether data met statistical assumptions?	☒ Yes, for at least one analysis<br>☐ No<br>☐ Not applicable
8	Experimental Animals	Are all species of animal used specified?	☒ Yes, for at least one experiment<br>☐ No
		Is the sex of the animals specified?	☒ Yes, for at least one experiment<br>☐ No<br>☐ Not applicable to species
		Is at least one of age, weight or developmental stage of the animals specified?	☒ Yes, for at least one experiment<br>☐ No
9	Experimental Procedures	Are both the timing and frequency with which procedures took place specified?	☒ Yes, for at least one experiment<br>☐ No
		Are details of acclimatisation periods to experimental locations provided?	☒ Yes, for at least one experiment<br>☐ No
10	Results	Are descriptive statistics for each experimental group provided, with a measure of variability (e.g. mean and SD, or median and range)?	☒ Yes, for at least one experiment<br>☐ No<br>☐ Not applicable to the type of data collected
		Is the effect size and confidence interval provided?	☐ Yes, for at least one experiment<br>☒ No<br>☐ Not applicable to the type of analysis used

Notes on questionnaire design

The ARRIVE guidelines are a useful resource for authors preparing manuscripts describing animal research, and also provide a framework to evaluate the transparency of those manuscripts. To assess reporting quality, numerous studies have in the past sought to operationalise reporting guidelines (including ARRIVE). Typically, this involves scoring a manuscript's degree of compliance with guideline items in a binary fashion (e.g. an item is either not reported or reported) [1-3], a graded fashion (e.g. not, partially, or completely reported) [4,5], or a combination of the two [6].

This questionnaire has been designed to be as concise and user-friendly as possible. The number of questions used to assess a manuscript's compliance has been kept to a minimum, and in most cases each question is designed to be answered in a binary fashion. Compliance with some Essential 10 sub-items is inherently impossible to judge in this way, instead requiring a subjective judgement on the level of detail provided. For this reason, not all sub-items are represented by a question in this questionnaire.

To facilitate binary answers, it has been necessary to identify the minimum information in a manuscript sufficient to comply with each question. The strengths of this approach include the relatively short length of the questionnaire (and the correspondingly low time burden of using it), and the avoidance of ambiguity that would arise from a graded answering system, in which an intermediate score (e.g. 'partially/insufficiently reported') could denote a number of distinct deficiencies in compliance with an item (e.g. either only part of the item was complied with, or only the reporting of some experiments in the manuscript complied with the item.)

Limitations of this approach centre on the necessity to identify the minimum information sufficient to comply with each question. In some cases, this has resulted in questions that require a guideline sub-item's criteria to have been fulfilled in the reporting of only one experiment in a manuscript. As a result, not all experiments in a manuscript may be described in a way that fulfils that criterion, despite the manuscript being considered to comply with the guidelines overall.

References

1. Hair *et al* (2020). *Res Integ Peer Rev*. doi: 10.1186/s41073-019-0069-3
2. Tihanyi *et al* (2019). *J Surg Res*. doi: 10.1016/j.jss.2018.10.038
3. Zhao *et al* (2020). *BMC Vet Res*. doi: 10.1186/s12917-020-02664-1
4. Han *et al* (2017). *Plos One*. doi: 10.1371/journal.pone.0183591
5. Chatzimanouil *et al* (2019). *J Am Soc Nephrol*. doi: 10.1681/ASN.2018050515
6. Leung *et al* (2018). *Plos One*. doi: 10.1371/journal.pone.0197882
